# Spatial Metabolomics and Transcriptomics Reveal Metabolic Reprogramming and Cellular Interactions in Nasopharyngeal Carcinoma with High PD-1 Expression and Therapeutic Response

**DOI:** 10.7150/thno.102822

**Published:** 2025-02-10

**Authors:** Lili Ji, Dujuan Wang, Guangzheng Zhuo, Zhe Chen, Liping Wang, Qian Zhang, Yuhang Wan, Guohong Liu, Yunbao Pan

**Affiliations:** 1 Department of Laboratory Medicine, Zhongnan Hospital of Wuhan University, Wuhan University, Wuhan 430071, China.; 2 Hubei Provincial Clinical Research Center for Molecular Diagnostics, Wuhan 430071, China.; 3 Department of Clinical Pathology, Houjie Hospital of Dongguan, The Affiliated Houjie Hospital of Guangdong Medical University, Dongguan 523960, China.; 4 Department of Otolaryngology Head and Neck Surgery, Zhongnan Hospital of Wuhan University, Wuhan 430071, China.; 5 Department of Radiology, Zhongnan Hospital of Wuhan University, Wuhan University, Wuhan 430071, China.; 6 Wuhan Research Center for Infectious Diseases and Tumors of the Chinese Academy of Medical Sciences, Wuhan 430071, China.

**Keywords:** nasopharyngeal carcinoma, spatial metabolomics, spatial transcriptomics, programmed death 1, tumor microenvironment

## Abstract

Nasopharyngeal carcinoma (NPC) is a heterogeneous cancer with variable therapeutic responses, highlighting the need to better understand the molecular factors influencing treatment outcomes. This study aims to explore spatially metabolic and gene expression alterations in NPC patients with different therapeutic responses and PD-1 expression levels.

**Methods:** This study employs spatial metabolomics (SM) and spatial transcriptomics (ST) to investigate significant alterations in metabolic pathways and metabolites in NPC patients exhibiting therapeutic sensitivity or elevated programmed death 1 (PD-1) expression. The spatial distribution of various cell types within the TME and their complex interactions were also investigated. Identified prognostic targets were validated using public datasets from TCGA, and further substantiated by *in vitro* functional analyses.

**Results:** SM analysis revealed substantial reprogramming in lipid metabolism, branched-chain amino acid (BCAA) metabolism, and glutamine metabolism, which were closely associated with therapeutic response and PD-1 expression. ST analysis highlighted the critical role of interactions between precursor T cells and malignant epithelial cells in modulating therapeutic response in NPC. Notably, six key genes involved in BCAA metabolism (*IL4I1, OXCT1, BCAT2, DLD, ALDH1B1, HADH*) were identified in distinguishing patients with therapeutic sensitivity from those with therapeutic resistance. Functional validation of DLD and IL4I1 revealed that gene silencing significantly inhibited NPC cell proliferation, colony formation, wound healing, and invasion. Silencing *DLD* or *IL4I1* induced cell cycle arrest. Reduction in α-Ketomethylvaleric acid (KMV) levels was demonstrated upon IL4I1 silencing. Immunohistochemical analysis further confirmed that high expression of these six genes was significantly associated with poor prognosis in NPC patients, a trend corroborated by data from the TCGA head and neck cancer cohort.

**Conclusions:** This study highlights the pivotal roles of key molecular players in therapeutic response in NPC, providing compelling evidence for their potential application as prognostic biomarkers and therapeutic targets, thereby contributing to precision oncology strategies aimed at improving patient outcomes.

## Introduction

Nasopharyngeal carcinoma (NPC) is a malignant tumor originating from the epithelial mucosa of the nasopharynx, predominantly prevalent in southern China, Southeast Asia, and North Africa. In 2022, over 120,000 new cases of NPC were reported globally, resulting in more than 70,000 deaths 1. The current standard treatments for NPC include cisplatin-based induction chemotherapy combined with concurrent chemoradiotherapy. Although NPC often exhibits high sensitivity to these treatments, a substantial proportion of patients experience recurrence and metastasis, leading to treatment failure and poor prognosis 2. In recent years, the advent of immunotherapy, particularly immune checkpoint inhibitors targeting the PD-1/PD-L1 pathway, has shown promise for recurrent and metastatic NPC 3. Nevertheless, the overall response rates remain suboptimal, highlighting the need for a deeper understanding of the tumor microenvironment (TME) and its metabolic underpinnings to improve therapeutic efficacy.

Metabolic reprogramming is a hallmark of cancer that enables tumor cells to sustain proliferation and evade immune surveillance even in nutrient-deprived conditions 4, 5. In NPC, metabolic alterations within the TME can influence immune cell function, promote immune evasion, and contribute to resistance to both chemotherapy and immunotherapy 5, 6. Traditional bulk metabolomics and transcriptomics approaches have provided valuable insights into metabolic and molecular changes in tumors; however, they fail to capture the spatial heterogeneity and complex cellular interactions within the TME. To address these limitations, spatially resolved technologies such as spatial transcriptomics (ST) and spatial metabolomics (SM) have emerged, offering unprecedented opportunities to explore the spatial architecture of tumors while preserving tissue context 7, 8.

The integration of ST and SM has been increasingly adopted in cancer research to elucidate the spatial interplay between gene expression and metabolic activity. Studies have demonstrated the utility of this approach in mapping metabolic heterogeneity and identifying spatially distinct tumor subpopulations in various cancer types 7, 8. However, its application in NPC remains limited, and the potential of combining these spatially resolved techniques to uncover the intricate relationship between metabolic pathways and treatment response in NPC has yet to be fully explored. Our study aims to bridge this critical gap by employing an integrated ST-SM approach to simultaneously visualize spatially resolved gene expression and metabolic alterations within NPC tissues. By constructing comprehensive spatial expression maps of genes and metabolites, our study seeks to identify key metabolic signatures associated with therapeutic sensitivity and PD-1 expression, providing novel insights into the metabolic-immune crosstalk within the NPC microenvironment.

This study represents a pioneering effort to leverage the combined power of ST and SM to advance our understanding of NPC pathophysiology. By offering a high-resolution spatial perspective on tumor metabolism and immune interactions, our findings have the potential to inform the development of novel therapeutic strategies and contribute to the optimization of personalized treatment approaches for NPC patients.

## Methods

### Sample Collection

We collected a total of 16 tissue samples, comprising 11 nasopharyngeal carcinoma (NPC) samples and 5 nasopharyngeal chronic inflammation with hyperplasia (CIH) samples, for spatial metabolomic (SM) analysis. The inclusion criteria were: Patients must have been pathologically confirmed to have NPC or CIH. Age between 20 and 80 years. Newly diagnosed patients who had not received any prior anti-tumor treatment. The exclusion criteria were: Patients with a history of systemic or local anti-tumor therapies. Presence of severe comorbidities or complications that could impact study outcomes or patient safety. Biopsy tissue that is too small to allow for adequate SM analysis. Inability to understand or comply with study procedures. Additionally, 8 samples (6 NPC and 2 CIH) were utilized for spatial transcriptomics (ST) sequencing. scRNA-seq was performed on samples from 9 patients to annotate cell types within the ST spots. Primary treatments included induction chemotherapy, immunotherapy, and concurrent chemoradiotherapy. Based on the Response Evaluation Criteria in Solid Tumours (RECIST 1.1), patients were classified into two groups: treatment-sensitive group (complete response [CR] and partial response [PR]) and treatment-resistant group (stable disease [SD] and progressive disease [PD]). Key genes were validated between tumor and normal tissues using data from the GEO database (GSE12452). A tissue microarray (TMA) constructed by our research group using a TMArrayer, comprising samples from 58 NPC patients, was utilized to validate the expression of several key biomarkers and their correlation with survival. Collected samples were stored at -80°C for subsequent ST and SM analysis.

### Immunohistochemistry

Tissue blocks were sectioned and deparaffinized using xylene and ethanol at varying concentrations, followed by antigen retrieval and blocking of endogenous peroxidase with 3% H_2_O_2_. Primary antibody incubation was conducted overnight at 4°C. Secondary antibody incubation and visualization were performed according to the protocol provided by the Department of Clinical Pathology of Houjie Hospital of Dongguan. Protein expression in the tissues was evaluated by clinical pathologist.

### Spatial Tissue Metabolomics Analysis

#### Section Preparation

NPC tissues were embedded in Cryo-Gel (Leica Microsystems, Germany), and sections approximately 10 μm thick were cut using a cryostat (Leica CM 1950, Leica Microsystems, Germany). Sections were mounted on positively charged slides (Thermo Fisher, USA) and stored at -80°C. Two sets of sections were used for mass spectrometry imaging (MSI) analysis. One set was stained with hematoxylin-eosin (H&E) and evaluated by a pathologist to annotate histological regions. The other set was used for ST analysis.

#### MSI Scanning Analysis

MSI analysis was performed at Oebiotech Technology Co., Ltd. The mass spectrometer resolution was set to 20,000, covering a mass range of 70-1200 Da. Samples were scanned continuously in the X direction at 0.2 mm/s, with a vertical step size of 100 μm in the Y direction.

#### Data Processing

Raw data files (.raw) were converted to "imzML" format using imzMLConverter and imported into MSiReader for analysis. Ion images were reconstructed using the Cardinal software package after background subtraction. All ion images were normalized using Total Ion Current (TIC) normalization for each pixel. Histological information from high-resolution HE images was projected onto the MSI data to extract tissue micro-regions.

Differentially expressed metabolites (DEMs) among groups were identified using orthogonal partial least squares discriminant analysis (OPLS-DA), with the Variable Importance in Projection (VIP) score assessing each metabolite's contribution to group differentiation. The significance of DEMs was further calculated using a two-tailed Student's t-test. Metabolic pathway enrichment analysis was conducted using the MetaboAnalyst package and the hypergeometric distribution test based on the Kyoto Encyclopedia of Genes and Genomes (KEGG) database.

Due to the unique data structure of metabolite quantitation, MS data for each pixel were clustered using Spatial Shrunken Centroids Clustering (SSCC) based on ion abundance. Metabolites were annotated using the pySM annotation framework 9 and the SmetDB metabolomics database (Lumingbio, Shanghai, China).

### Spatial Transcriptomics Sequencing

Spatial Transcriptomics Sequencing analysis was performed at Oebiotech Technology Co., Ltd. Frozen tissue sections were fixed onto 10X Visium CytAssist spatial slides, followed by methanol fixation, HE staining, image scanning, and tissue decolorization according to the 10x Genomics protocol (CG000495). The DNA library was sequenced using the PE-150 mode for high-throughput sequencing. High-throughput sequencing generated raw data in fastq format. We processed Visium ST sequencing data and brightfield microscopy images using Space Ranger software (version 2.0.1) from 10x Genomics. This process involved detecting the capture area of the tissue on the chip, aligning it to the reference genome (human: GRCh38), and assigning reads to specific spots based on spatial barcode information. The quality of samples was assessed by evaluating the total number of spots, the number of reads per spot, the number of detected genes, and the count of unique molecular identifiers (UMIs).

### Data Processing

Data processing was carried out using the Seurat package (version 4.1.0). The sctransform function was applied to construct a regularized negative binomial model of gene expression for normalization and scaling. Highly variable genes (HVGs) were identified using the FindVariableGenes function, focusing on the top 3000 HVGs. Dimensionality reduction was performed through principal component analysis (PCA) based on these HVGs. Batch effects were corrected using the Harmony algorithm. Unsupervised clustering and visualization were conducted using the FindNeighbors, FindClusters, and RunUMAP functions, and results were displayed using UMAP plots. Gene set variation analysis (GSVA) was performed for pathway enrichment, followed by differential pathway analysis using the lmFit function from the limma package.

### Spatial Cell Annotation

Cell types were annotated using the RCTD package (version 1.1.0), which assigns cell types to ST spots. Single-cell sequencing was performed at Singleron Technology Co., Ltd and annotated scRNA-seq data from NPC patients served as a reference for defining cellular composition within each ST spot. Spots with at least one UMI and each cell type with at least one cell were retained. The create.RCTD function was executed with default parameters, and the run.RCTD function was executed with doublet_mode set to FALSE.

### Pseudotime Trajectory Inference (PST)

Pseudotime trajectory inference was conducted using stLearn^10^. Gene expression in the spots was initially standardized using stSME normalization, followed by SME clustering analysis. The PST algorithm identified both local and global hierarchical relationships, computing pseudotime using the diffusion pseudotime (DPT) method. The root node was defined based on biological significance, and pseudo-space-time distance (PSTD) was calculated by integrating gene expression values with physical distances.

### Spatial Transcriptomics Spot Distance-Based Analyses

Spatial co-localization of cell types was analyzed using spatial transcriptomics spot distance-based analyses. A specific cell type was selected as a reference point, and Euclidean distances from all spots to this reference point were calculated. Cell type density curves were plotted based on these distances to examine colocalization at various ranges. Distances were categorized into four tiers: reference (0), start (1/3 quantile distance), middle (2/3 quantile distance), and end (farthest distance), with spatial images generated accordingly.

### Spatial Ligand-Receptor Analysis

Spatial ligand-receptor interactions among all cell types in the ST data were analyzed using stLearn (v0.4.11). The st.tl.cci.run function identified significant ligand-receptor interactions, excluding pairs expressed in fewer than 20 spots or with an adjusted p-value greater than 0.05. The st.tl.cci.run_cci function inferred cell-cell interaction relationships based on cell type abundance in the spots and significant ligand-receptor pairs, with results visualized using heatmaps.

### Cell Culture

NPC cell lines NPC43 and SUNE1 were cultured in an incubator at 37°C with 5% CO₂. The cells were maintained in RPMI 1640 medium (Gibco) supplemented with 8% fetal bovine serum (FBS) and 1% penicillin-streptomycin.

### siRNA Transfection

Cells were seeded in 6-well plates at a density of 200,000 cells per well. siRNA and transfection reagent (Lipofectamine 3000) were mixed according to the manufacturer's protocol and incubated at room temperature for 20 minutes. The transfection mixture was then gently added to the cells. After 6-8 hours of incubation, the medium was replaced with fresh complete medium, and the cells were cultured for an additional 24-48 hours.

### RNA Extraction and Quantitative Real-Time PCR (qRT-PCR)

Total RNA was extracted using TRIzol reagent (Life Technologies) following the manufacturer's instructions. Reverse transcription was performed using reverse transcriptase (TOYOBO). Quantitative real-time PCR was conducted using an UltraSYBR Mixture kit (CWBIO) on a Tianlong qPCR system. Fold changes in gene expression were calculated based on the supplier's protocol.

### CCK-8 Cell Proliferation Assay

Cells were seeded in 96-well plates at a density of 1,000-2,000 cells per well and incubated for 24 hours before treatment. After treatment, 10 μL of CCK-8 solution was added to each well, followed by a 3-hour incubation. Absorbance was measured at 450 nm using a microplate reader. Cell proliferation rates were calculated based on absorbance values, and growth curves were plotted.

### Colony Formation Assay

Cells were seeded in 6-well plates at a density of 1,000 cells per well and cultured for 10-14 days until visible colonies formed. Colonies were fixed, stained, and those containing more than 50 cells were counted under a microscope.

### Wound Healing Assay

Cells were seeded in 6-well plates at a density of 200,000 cells per well. A straight scratch was made in the cell monolayer using a pipette tip to create a wound. Initial images of the wound were captured under a microscope, and subsequent images were taken after 48 hours to measure wound width.

### Transwell Invasion Assay

Transwell chambers were pre-coated with Matrigel. Cells were seeded into the upper chamber at a density of 20,000 cells per well. After 48 hours of incubation, cells on the upper surface of the membrane were removed. The invaded cells on the lower surface were fixed with paraformaldehyde, stained with crystal violet, and counted under a microscope.

### Cell Cycle Analysis

Cells were harvested and fixed in 70% ethanol overnight. After washing with cold PBS, cells were resuspended in propidium iodide (PI) staining solution and incubated in the dark for 30 minutes. Cell cycle distribution was analyzed using a flow cytometer (BD FACSCanto), and the proportions of cells in G1, S, and G2 phases were calculated.

### α-Ketomethylvaleric Acid (KMV) Measurement

The concentration of α-Ketomethylvaleric acid was determined using an enzyme-linked immunosorbent assay (ELISA) kit (FANKEW) according to the manufacturer's protocol. Absorbance was measured at 450 nm, and metabolite levels were calculated using a standard curve.

### Statistical Analysis

All statistical analyses were performed using R (version 4.3.2) and GraphPad Prism (version 9.0). Differential expression analysis between groups was conducted using the FindMarkers function in Seurat, employing the Wilcoxon test. Differentially expressed genes were filtered based on a fold change greater than 1.5 and a p-value less than 0.05. Pearson correlation coefficients were calculated using the cor function in R, and correlation heatmaps were generated using the pheatmap package. Group differences were analyzed using the Wilcoxon test. Violin plots and spatial feature plots were created using Seurat's VlnPlot and SpatialFeaturePlot functions. Survival and ROC curves were plotted using GraphPad Prism. Box plots and bar charts were drawn using two-tailed Student's t-tests in Prism. Gene Ontology (GO) enrichment analysis plots were generated online using Metascape (http://metascape.org). Statistical significance was defined as p < 0.05, with * for p < 0.05, ** for p < 0.01, *** for p < 0.001, **** for p < 0.0001.

## Results

### Investigating the NPC Tumor Microenvironment Using Spatial Multi-Omics

**Figure 1:**
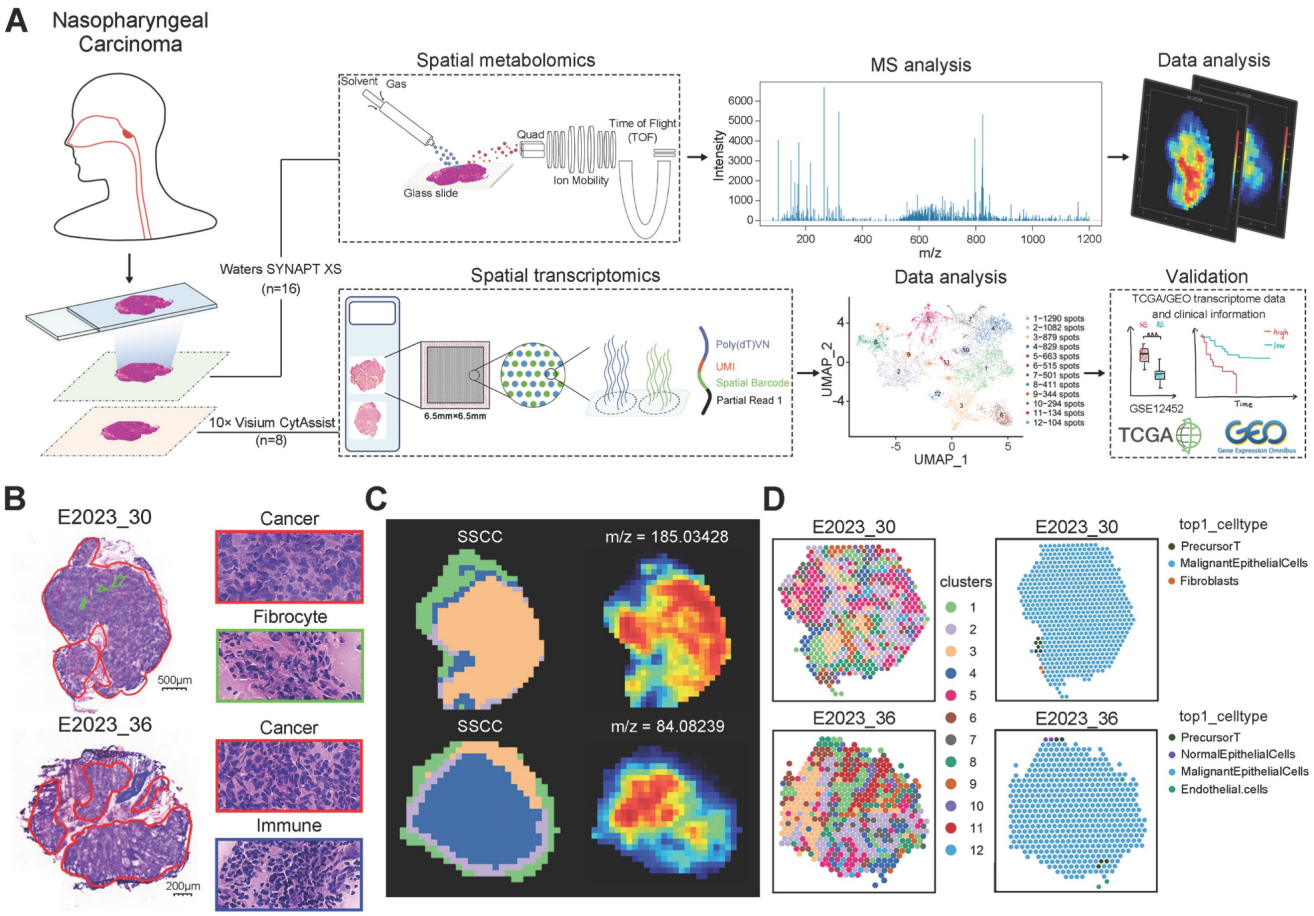
** Data Analysis Workflow and Spatial Analysis.** A: Schematic representation of the workflow for spatial transcriptomics (ST) and spatial metabolomics (SM) analysis in NPC. B: HE stained images of tissue sections from patients No. 30 and No. 36, highlighting distinct pathological regions. C: Spatial Shrunken Centroids Clustering (SSCC) of all pixels from two NPC samples (left). Ion images for m/z 185.03428 and 84.08239 (right), with colors ranging from blue (low intensity) to red (high intensity). D: Unsupervised clustering of all spots in tissue sections from two patients, resulting in 12 clusters and their spatial distribution (left). Right: Annotation of cell composition in spatial spots using the RCTD algorithm, indicating the predominant cell type per spot.

To investigate metabolic reprogramming and intercellular interactions within the NPC tumor microenvironment, we utilized spatial multi-omics techniques to identify key molecular events influencing therapeutic response. Fresh tissue samples were obtained from 11 clinically diagnosed NPC cases and 5 cases diagnosed with chronic inflammatory hyperplasia (CIH) as controls. The clinical information of the patients is shown in Table S1. Frozen sections were prepared, with adjacent sections subjected to spatial metabolomics analysis using the Waters SYNAPT XS, and spatial transcriptomics (ST) analysis was performed on 8 samples (6 NPC and 2 CIH) using the 10× Visium CytAssist platform, following the workflow in Figure 1A. Hematoxylin and eosin (HE) images from two patients (Figure 1B) revealed the pathological composition of tumor regions, fibroblast regions, and immune regions. Additional histological images (Figure S1A) indicated mixed tumor-immune regions and normal epithelial regions, reflecting the heterogeneity among NPC patients.

We applied Spatial Shrunken Centroids Clustering (SSCC) based on ion abundance at each pixel. Clustering results are shown in Figure 1C (left), where similarly colored regions represent similar expression levels. Imaging analysis of all m/z values (Figure 1C, right) provided expression information across tissue sections, with redder colors indicating higher ion abundance.

ST analysis detected 7,046 spots across all samples, with average UMI counts per spot ranging from 31,879 to 97,263, and average gene counts per spot from 6,456 to 10,605 (Figure S1B-C). After normalizing the data with sctransform, Principal Component Analysis (PCA) was conducted for dimensionality reduction. Batch effects were removed using the harmony algorithm, and clustering was visualized using Uniform Manifold Approximation and Projection (UMAP) (Figure S1D). Dimensionality reduction and clustering yielded 12 distinct clusters (Figure 1D, left). To further explore cell-cell interactions within the NPC microenvironment, we applied the robust cell type decomposition (RCTD) algorithm, utilizing scRNA-seq data from 9 samples as a reference dataset for spatial mapping, allowing us to infer cell type composition at each spot (Figure 1D, right).

### Metabolic Reprogramming Associated with Therapeutic Response and PD-1 Expression

**Figure 2:**
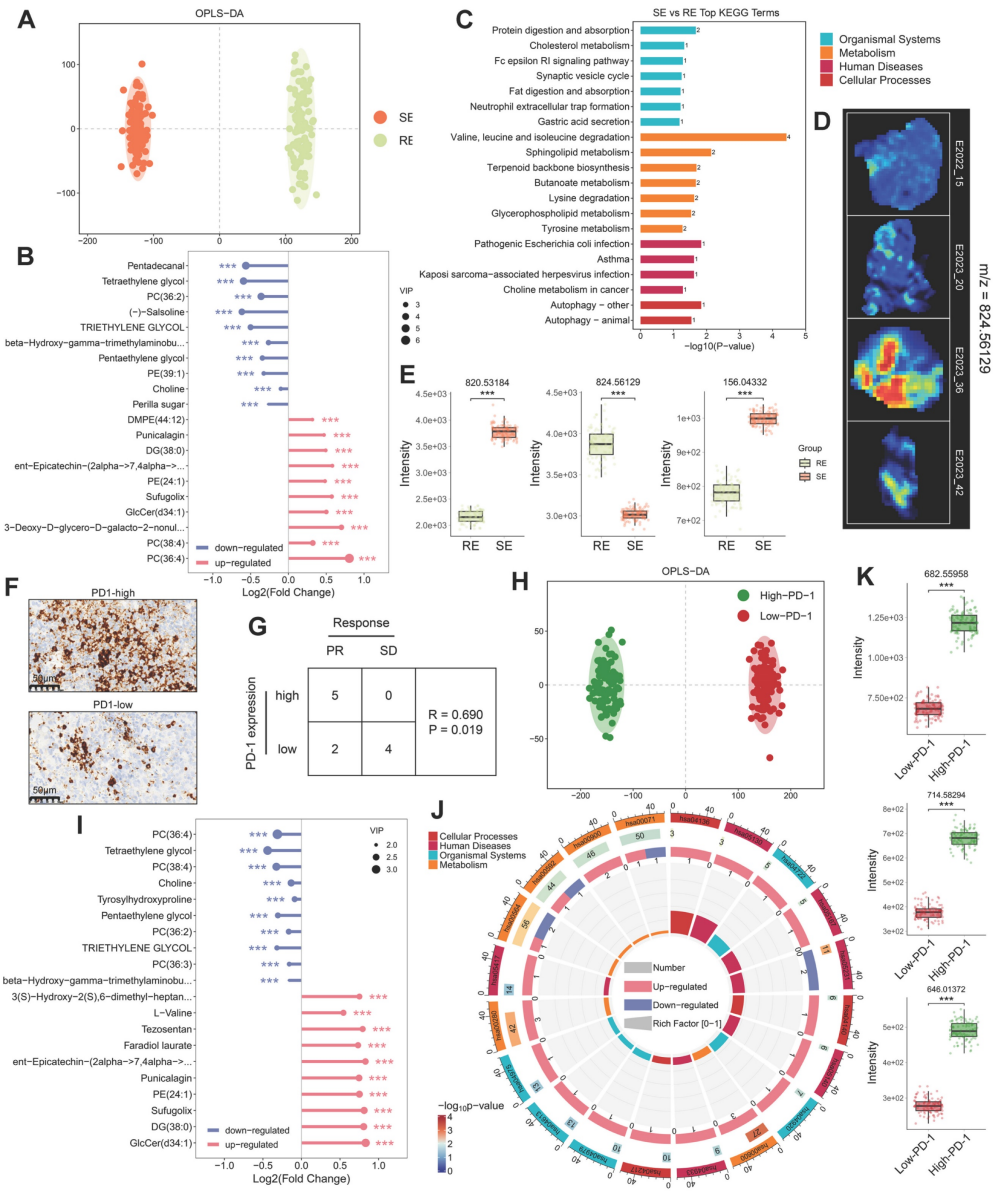
** Metabolic Differences and Treatment Response Analysis.** A: OPLS-DA score plot differentiating treatment-sensitive and resistant sample pixels, with ellipses representing 95% confidence intervals. B: Lollipop chart illustrating differentially expressed metabolites between treatment response groups, with the x-axis indicating log2(Fold Change) and the y-axis representing differential metabolites. The dot size reflects VIP value, with red indicating upregulation and blue downregulation. C: KEGG pathway enrichment analysis of differentially expressed metabolites, displaying the top 20 pathways with the smallest p-values. The x-axis represents -log10(p-value), and the y-axis lists different pathways. Colors represent pathway categories, and bar labels indicate the number of enriched metabolites per pathway. D: Spatial ion images of m/z 824.56129 in four samples. E: Boxplots of three selected top 50 VIP metabolites enriched in significant pathways, with the y-axis representing ion intensity. F: Immunohistochemical visualization of PD-1 expression in NPC tissue sections, categorizing samples into PD-1-high and PD-1-low groups. G: A 2×2 contingency table is presented showing the relationship between PR (partial response) and SD (stable disease) samples, and samples with high and low PD-1 expression. Spearman correlation was calculated using SPSS. H: OPLS-DA score plot differentiating PD-1-high and PD-1-low sample pixels, with ellipses representing 95% confidence intervals. I: Lollipop chart showing the top 10 upregulated and downregulated metabolites in PD-1-high and PD-1-low groups. J: Circular plot of KEGG enrichment analysis for PD-1 group differentially expressed metabolites. The four concentric circles represent, from outer to inner: pathway classification, background metabolism pathway count, number of upregulated (red) and downregulated (blue) metabolites, and Rich Factor value for each pathway, with gridlines marking 0.2 increments. K: Boxplots comparing expression levels of three top 50 VIP metabolites enriched in significant pathways across different groups. RE: treatment-resistant. SE: treatment-sensitive.

We characterized the NPC spatial metabolome using the DESI platform (resolution: 100 μm × 100 μm) (Figure S2A). To investigate metabolic differences between NPC patients with varying therapeutic responses, we performed OPLS-DA multivariate statistical analysis, identifying differential metabolites (DEMs). Samples from treatment-sensitive and treatment-resistant groups exhibited distinct clustering and separation trends (Figure 2A). The Variable Importance in Projection (VIP) values derived from the OPLS-DA model were used to identify potential biomarkers. To avoid overfitting, we validated the model using 7-fold cross-validation and response permutation testing (RPT), with R2 and Q2 values indicating no overfitting and good predictive power (Figure S2B). Based on the OPLS-DA V-plots, we selected ions with VIP > 1, p < 0.05, and FoldChange > 1.2, leading to the identification of 169 significant DEMs, which were visualized using a lolipopmap (Figure 2B). KEGG pathway enrichment analysis of the selected DEMs highlighted significant pathways such as valine, leucine, and isoleucine degradation, sphingolipid metabolism, and glycerophospholipid metabolism (Figure 2C), indicating a potential link between BCAA and lipid metabolism and therapeutic response. Ion imaging (Figure 2D) of the top 50 DEMs enriched in significant KEGG pathways and box plots comparing groups (Figure 2E) further supported these findings.

Immunohistochemistry (IHC) was used to classify samples into PD-1-high and PD-1-low groups (Figure 2F). Through correlation analysis, we found that the treatment response was correlated with the expression level of PD-1 (Figure 2G). Therefore, we further analyzed the metabolic reprogramming associated with high and low PD-1 expression. OPLS-DA results demonstrated clear clustering for PD-1-high and PD-1-low samples (Figure 2H). Based on VIP > 1, FoldChange > 1.2, and p < 0.05, 222 DEMs were identified (Figure 2I). KEGG pathway enrichment analysis of these DEMs identified the top 20 pathways with the smallest p-values, visualized in a KEGG enrichment circle plot (Figure 2J). Pathways such as valine, leucine, and isoleucine degradation (has00280) and glycerophospholipid metabolism (has00540) were also altered, further suggesting that BCAA and lipid metabolism are related to both therapeutic response and PD-1 expression levels. The top 50 DEMs from the PD-1 grouping and some metabolic ions enriched in pathways are shown in Figure 2K.

### Relationship Between Fatty Acid Metabolism, Therapeutic Response, and PD-1 Expression

**Figure 3:**
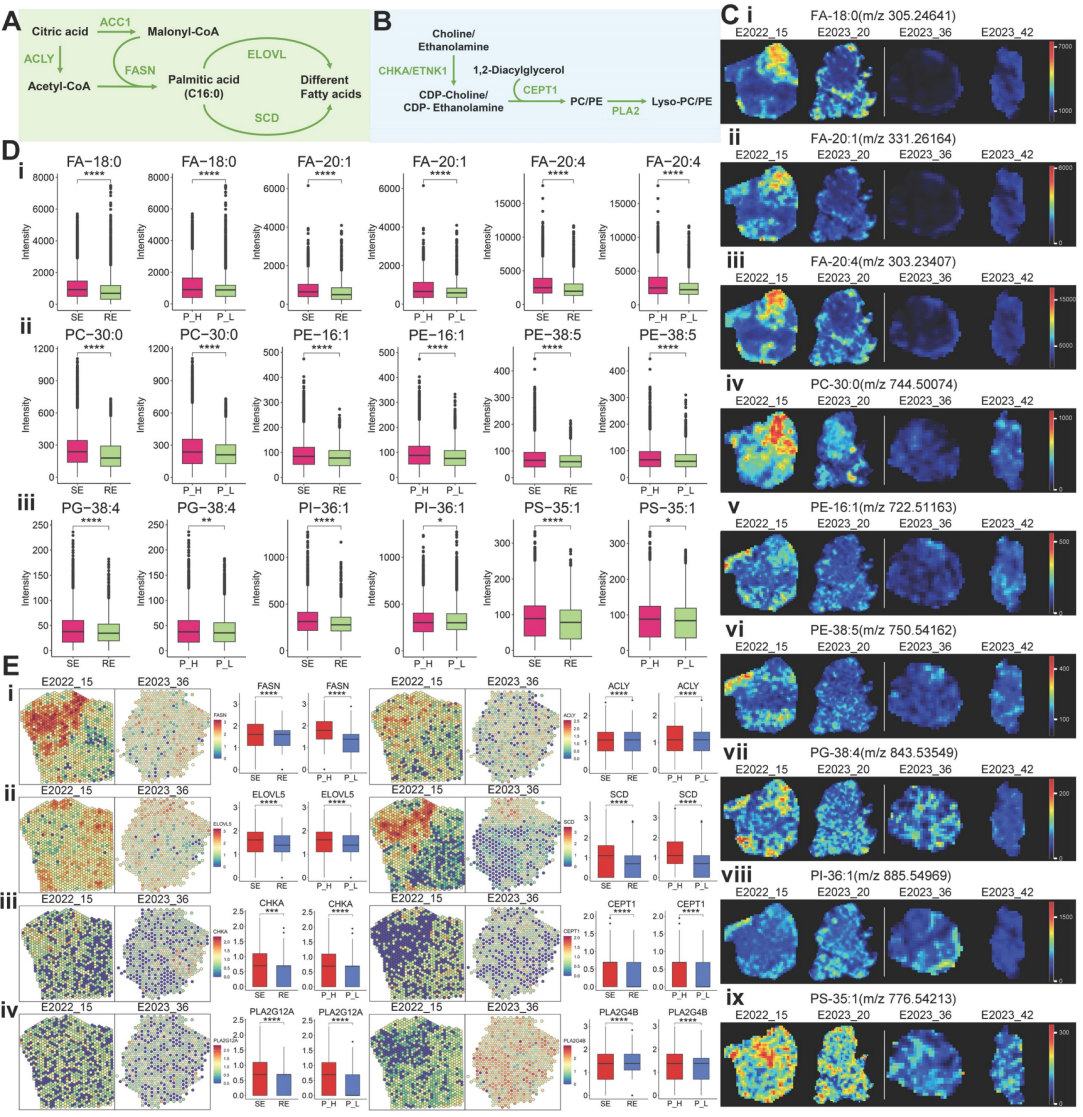
** Lipid Metabolism Pathways and Spatial Expression Analysis.** A: Schematic diagram of the fatty acid synthesis pathway. B: Schematic diagram of phosphatidylcholine and phosphatidylethanolamine synthesis and degradation pathways. C: MS images of various lipids in four samples, with colors from blue (low intensity) to red (high intensity). D: Boxplots comparing lipid expression levels between two groups (treatment-sensitive vs. resistant, PD-1-high vs. low), analyzed using the Wilcoxon test. E: Spatial expression images of key genes in the fatty acid synthesis, phosphatidylcholine, and phosphatidylethanolamine synthesis pathways in two patients (left), with corresponding boxplots comparing expression levels in two groups. Colors range from blue (low expression) to red (high expression). RE: treatment-resistant. SE: treatment-sensitive. P_H: PD-1 high. P_L: PD-1 low.

Fatty acids (FAs) are crucial for cancer proliferation and signaling 11. Under the action of ATP citrate lyase (ACLY) and acetyl-CoA carboxylase 1 (ACC1), citrate generates precursor molecules for long-chain fatty acids: acetyl-CoA and malonyl-CoA. These are synthesized into palmitic acid (FA-16:0) by fatty acid synthase (FASN) and further modified by elongation of very long-chain fatty acids protein (ELOVL) and stearoyl-CoA desaturase (SCD) to produce various FAs (Figure 3A). Using ST and SM techniques, we observed alterations in FA metabolism pathways in different therapeutic response groups. Representative FA mass spectrometry imaging (MSI) maps (Figure 3C (i-iii)) showed higher FA expression in treatment-sensitive and PD-1-high samples (No. 15, No. 20) compared to treatment-resistant and PD-1-low samples (No. 36, No. 42) (Figure 3D (i)), indicating a potential association between FA metabolism and therapeutic response. Consistently, FASN and ACLY expression was elevated in treatment-sensitive and PD-1-high samples, as confirmed by spatial expression and comparative analysis (Figure 3E (i)). Moreover, ELOVL and SCD, involved in FA chain elongation and desaturation, were also upregulated in treatment-sensitive and PD-1-high samples (Figure 3E (ii)).

Phospholipids, essential components of cell membranes, can influence therapeutic response by regulating drug efflux pumps and serve as potential therapeutic targets 12. We examined the spatial imaging of phosphatidylcholines (PC), phosphatidylethanolamines (PE), phosphatidylglycerols (PG), phosphatidylinositols (PI), and phosphatidylserines (PS) (Figure 3C (iv-ix)). Comparative analysis indicated increased expression of most phospholipids in treatment-sensitive and PD-1-high samples (Figure 3D (ii, iii)). In the phospholipid metabolism pathway, choline and ethanolamine are converted into CDP-choline and CDP-ethanolamine through choline kinase alpha (CHKA) and ethanolamine kinase 1 (ETNK1), respectively. These then combine with diacylglycerol to form PC and PE via choline/ethanolamine phosphotransferase 1 (CEPT1). Subsequently, PC/PE is hydrolyzed into Lyso-PC/PE by phospholipase A2 (PLA2) (Figure 3B). Genes such as *CHKA, CEPT1, PLA2G12A,* and* PLA2G4B* were upregulated in treatment-sensitive and PD-1-high samples (Figure 3E (iii, iv)), suggesting that more active lipid metabolism in these samples may contribute to enhanced cancer therapeutic efficacy.

### Amino Acid Metabolism Associated with Treatment Response

**Figure 4:**
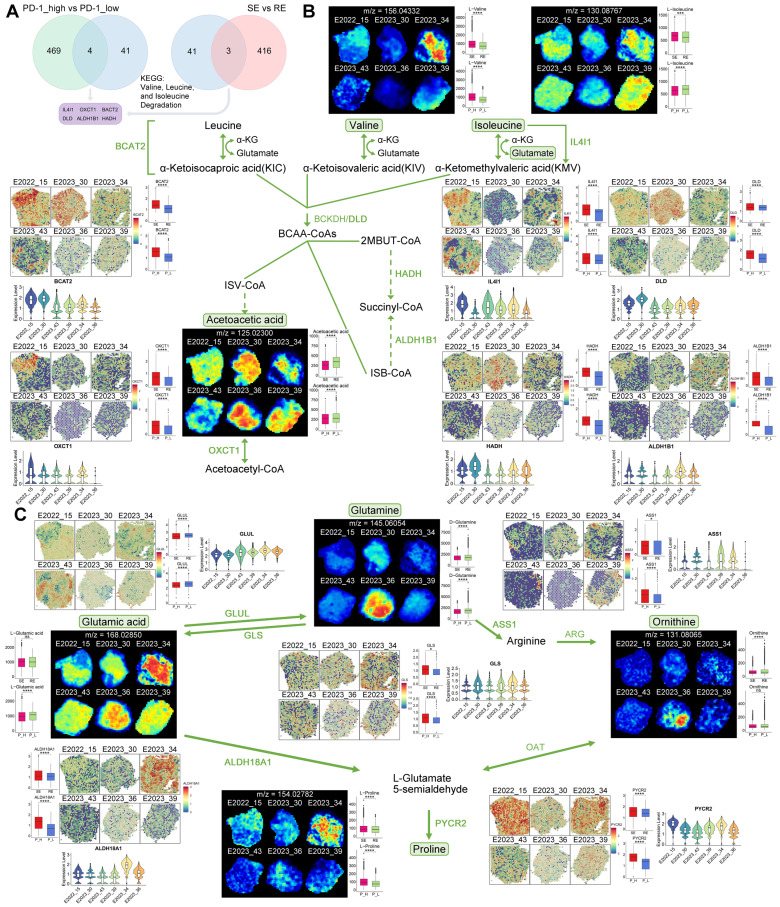
** Key Gene and Metabolite Analysis in Metabolic Pathways.** A: Selection process for six key genes (IL4I1, OXCT1, BACT2, DLD, ALDH1B1, HADH). B: MS images of key metabolites in the BCAA metabolic pathway, with boxplots comparing their expression between two groups. Also shown are spatial expression images of key genes (colors from blue to red indicate expression levels) and violin plots of gene expression levels across different samples. C: MS images of key metabolites in the glutamine metabolism pathway, with boxplots comparing their expression between two groups. Additionally, spatial expression images of key genes, boxplots comparing gene expression between two groups, and violin plots of gene expression levels across different samples are presented. RE: treatment-resistant. SE: treatment-sensitive. P_H: PD-1 high. P_L: PD-1 low.

To explore the relationship between metabolic pathway alterations and treatment response, we performed differential gene expression analysis using spatial transcriptomics (ST) data. Six key genes—*IL4I1*, *OXCT1*, *BCAT2*, *DLD*, *ALDH1B1*, and *HADH*—were identified as potentially associated with treatment response and PD-1 expression (Figure 4A).

A schematic diagram of the BCAA metabolism pathway centered on these key genes is shown in Figure 4B. Free BCAAs (leucine, valine, and isoleucine) undergo transamination by branched-chain amino acid transaminase 2 (BCAT2), transferring nitrogen to α-ketoglutarate (α-KG) to produce glutamate and corresponding branched-chain keto acids—alpha-ketoisocaproic (KIC), alpha-ketoisovaleric (KIV), and alpha-keto-beta-methylvaleric (KMV). These keto acids are then metabolized by branched-chain keto acid dehydrogenase (BCKDH) to yield acyl-CoA derivatives. Subsequently, HADH catalyzes acyl-CoA derivatives (2-methylbutanoyl-CoA [2MBUT-CoA]), leading to the production of propionyl-CoA, which is further converted into succinyl-CoA by methylmalonyl-CoA mutase, eventually entering the tricarboxylic acid (TCA) cycle (Figure 4B).

Utilizing spatial metabolomics data, we conducted ion imaging of metabolites and presented box plots to compare their levels across different sample groups. In parallel, we used spatial transcriptomics (ST) data to visualize the spatial expression of key enzymes, displaying violin plots of gene expression and box plots illustrating group differences (Figure 4B). Notably, valine and isoleucine were highly expressed in the treatment-sensitive group, whereas acetoacetic acid demonstrated an inverse pattern (Figure 4B). Enzymes involved in BCAA metabolism, such as BCAT2, IL4I1, DLD, HADH, ALDH1B1, and OXCT1, were upregulated in treatment-sensitive and PD-1-high samples (Figure 4B), indicating that active BCAA metabolism may be linked to therapeutic responsiveness.

Further, we examined glutamine-related metabolic reprogramming within the BCAA pathway. Glutamine plays a vital role in providing carbon and nitrogen for tumor metabolism 13. We mapped key metabolites and regulatory genes throughout the glutamine metabolic pathway (Figure 4C). Glutamine levels were lower in treatment-sensitive and PD-1-high samples, while glutaminase (GLS), the enzyme catalyzing the conversion of glutamine to glutamic acid, was upregulated in these same samples, suggesting a potential connection between glutamine catabolism, treatment response, and PD-1 expression (Figure 4C). Additionally, glutamate-ammonia ligase (GLUL), responsible for converting glutamic acid to glutamine, exhibited reduced expression in treatment-sensitive and PD-1-high samples (Figure 4C).

The glutamine-related pathway also encompasses the synthesis of arginine and proline, which are interlinked within metabolic pathways 14. The synthesis of arginine is regulated by argininosuccinate synthase 1 (ASS1), which was found to be upregulated in treatment-sensitive and PD-1-high samples, corresponding with the observed decrease in glutamine levels (Figure 4C). Proline can be synthesized by aldehyde dehydrogenase 18 family member A1 (ALDH18A1) and pyrroline-5-carboxylate reductase (PYCR) 15. Our ST data showed increased expression of ALDH18A1 and PYCR2 in treatment-sensitive and PD-1-high samples, along with elevated proline levels (Figure 4C), suggesting that enhanced arginine and proline synthesis may contribute to better therapeutic outcomes.

### Molecular Pathways and Cell Types Associated with Treatment Response

**Figure 5:**
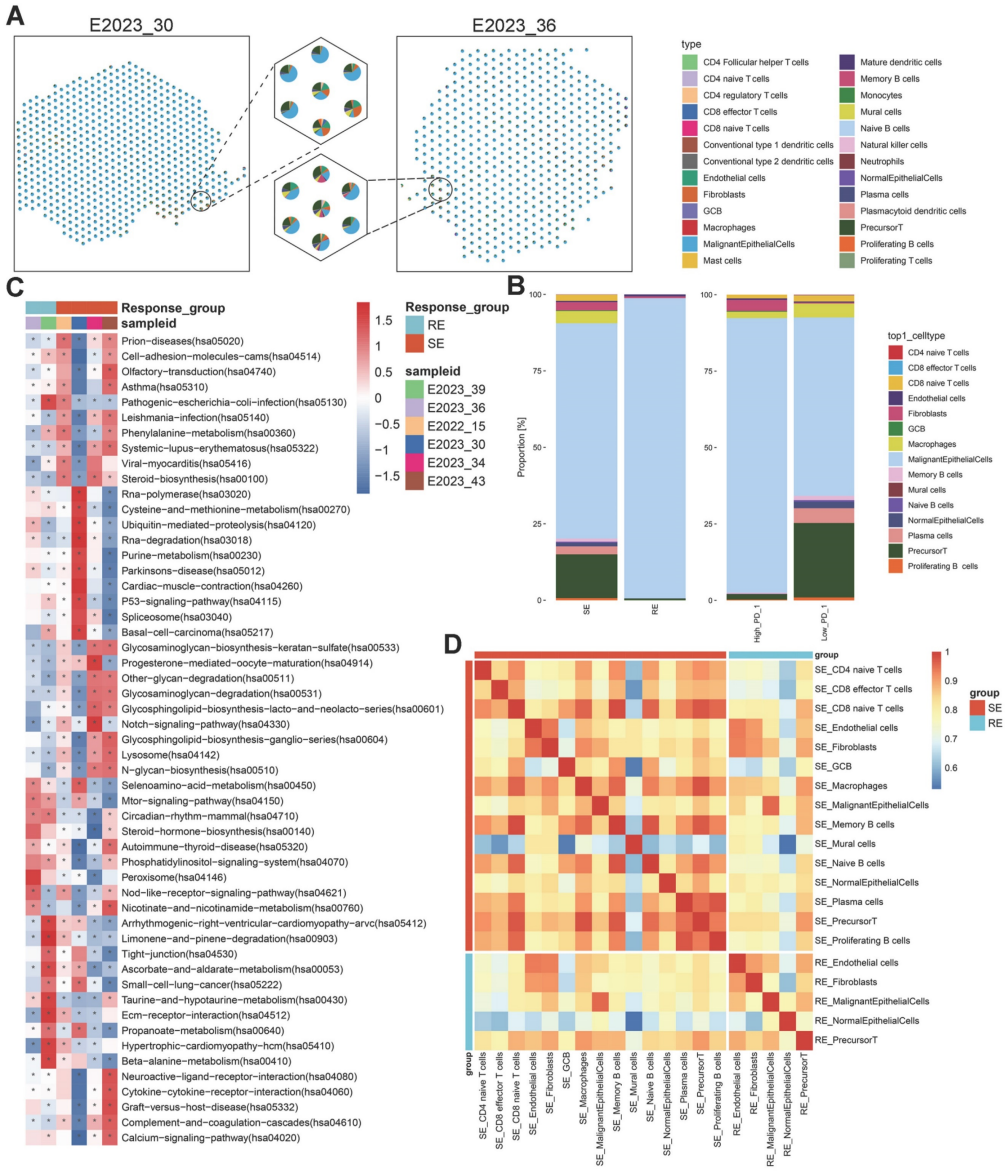
** Cell Type Composition and Enrichment Analysis.** A: Pie chart depicting the composition of cell types identified in various spots on spatial tissue sections, with different colors representing different cell types. B: Stacked bar charts illustrating the relative proportions of annotated subpopulations in treatment-sensitive vs. resistant samples and PD-1-high vs. low samples. C: GSVA enrichment analysis of all spots in spatial tissue sections from six NPC samples, shown as a heatmap of signaling pathways. The background gene set is the KEGG database. The x-axis represents sample names and groups, and the y-axis represents metabolic entries. D: Heatmap of cell type correlations (Pearson coefficient) by group, with both axes representing cell types. RE: treatment-resistant. SE: treatment-sensitive.

To explore transcriptomic differences between treatment-sensitive and treatment-resistant samples, we generated a spatial transcriptomics (ST) atlas. This atlas depicted the distribution of cell types across samples (Figure 5A). The predominant cell type in these samples was malignant epithelial cells, followed by precursor T cells, underscoring their significant roles in therapeutic response (Figure S2C). A comparative analysis of major cell types across different tumor groups revealed a higher proportion of precursor T cells and macrophages in treatment-sensitive samples compared to treatment-resistant ones (Figure 5B). Precursor T cells were more abundant in PD-1-low samples, followed by macrophages, plasma cells, CD8 naïve T cells, and progenitor T cells, suggesting a potential relationship between these cells, PD-1 expression, and therapeutic outcomes.

To pinpoint molecular pathways associated with therapeutic response, we applied gene set variation analysis (GSVA) on the ST data and visualized pathway enrichment using the KEGG database (Figure 5C, Figure S3A). Treatment-sensitive samples exhibited significant enrichment in metabolic pathways, such as phenylalanine metabolism, purine metabolism, and glycosphingolipid biosynthesis, aligning with the lipid metabolism reprogramming identified through spatial metabolomics data. Conversely, treatment-resistant samples showed enrichment in other metabolic pathways, including ascorbate and aldarate metabolism, taurine and hypotaurine metabolism, and beta-alanine metabolism. PD-1-high samples were enriched in purine metabolism and glycosphingolipid biosynthesis, while PD-1-low samples shared enrichment patterns with treatment-resistant samples. These results suggest that therapeutic sensitivity and PD-1 expression are linked to the regulation of metabolic pathways, particularly lipid metabolism.

To further investigate the observed differences in precursor T cells and macrophages within the tumor microenvironment across different groups, we analyzed the correlations between various cell types and presented these interactions in heatmaps (Figure 5D, Figure S3B). The analysis revealed strong correlations between macrophages, precursor T cells, and CD8 naïve T cells, indicating that these interactions may significantly influence the immune response within the tumor microenvironment and, consequently, affect therapeutic outcomes.

### Cell Differentiation and Intercellular Interactions Related to Therapeutic Response

**Figure 6:**
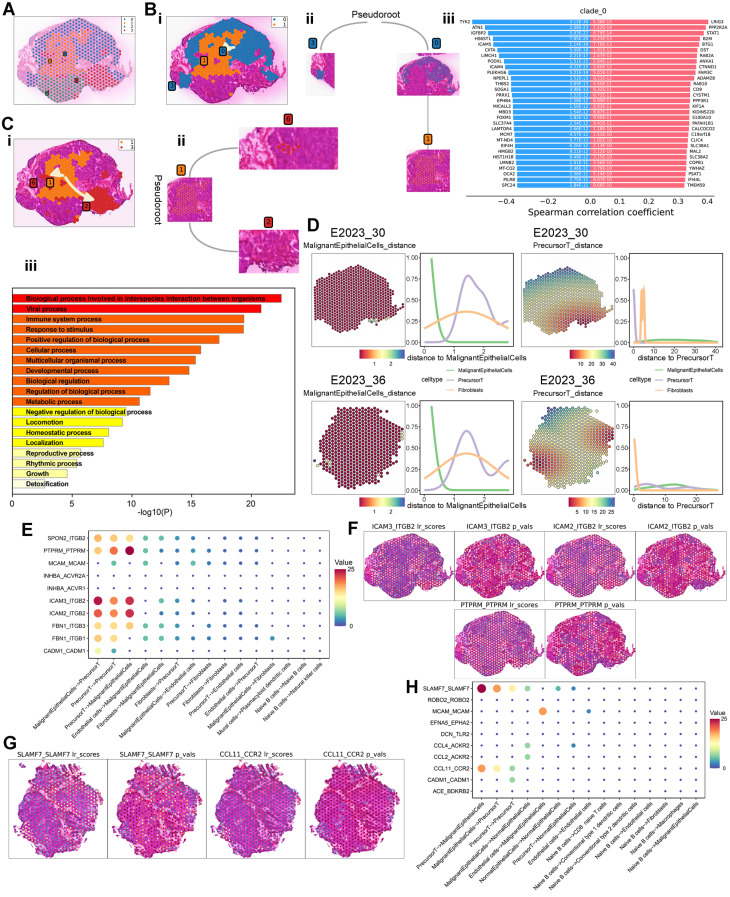
** Spatial Trajectory Inference and Cell Interaction Analysis.** A: Global clustering of all spots by stSME into four clusters. B: i) Spatial trajectory inference visualization from cluster 0 to cluster 1. ii) Directional tree diagram of the differentiation trajectory from cluster 0 to cluster 1. iii) Differential expression of transition marker genes along the spatial trajectory, with red indicating upregulated and blue downregulated genes. C: i) Spatial trajectory inference visualization from cluster 1 to cluster 3. ii) Directional tree diagram of the differentiation trajectory from cluster 1 to cluster 3. iii) Metascape enrichment analysis of transition gene sets, with the x-axis representing -log10(p-value). D: Left: Euclidean distance between malignant epithelial cells or precursor T cells and surrounding cells. Right: Density analysis of cell types at varying distances from malignant epithelial cells or precursor T cells. E: Top 10 L-R pairs by interaction quantity between different cell types, with colors from blue to red representing interaction quantity. F-G: Scores for significantly expressed L-R pairs and their spatial distribution, with each spot's scores representing interaction strength with neighboring tissue. H: Top 10 L-R pairs by interaction quantity between different cell types.

To further investigate the spatially resolved molecular mechanisms and cell types associated with therapeutic response, we analyzed the spatial transcriptomic characteristics within NPC samples. We selected 558 spots from NPC samples, identifying cancer and fibrocyte regions using Loupe software based on HE-stained pathological areas, for stLearn pseudotemporal analysis. These spots were categorized into four parent clusters (Figure 6A). Based on their spatial distribution in the HE images and biological relevance, we defined two pseudotemporal trajectories: 0→1 (peritumoral → intratumoral) and 1→3 (intratumoral → peritumoral). Pseudotemporal (PST) analysis was conducted according to stSME clustering results, allowing us to infer differentiation trajectories between specific subclusters (Figure 6Bi, Ci). The subcluster differentiation trajectories are depicted in dendrograms (Figure 6Bii, Cii). Additionally, we identified transition genes along these trajectories based on differential gene expression (Figure 6Biii, Figure S3C) and performed enrichment analysis using Metascape. This analysis revealed significant shifts in metabolic processes and immune system activities as cells differentiated from peritumoral to intratumoral regions (Figure S3D). Similarly, from intratumoral to peritumoral regions, the enrichment analysis indicated changes in immune system processes, metabolic pathways, viral activity, and other biological processes (Figure 6Ciii), suggesting alterations in metabolism and immune responses during cell differentiation.

To explore intercellular interactions within NPC, we utilized spatial transcriptomics (ST) to assess the co-localization of different cell types. By using spots corresponding to malignant epithelial cells and precursor T cells as reference points, we calculated Euclidean distances (Figure S3E) and plotted cell type density curves (Figure 6D). In a treatment-sensitive sample (No.30), a notable change in precursor T cell distribution was observed with increasing distance from malignant epithelial cells, exhibiting a rise-then-fall pattern. This pattern was similar to the overall distribution change of precursor T cells in a treatment-resistant sample (No. 36). However, using precursor T cells as the reference point revealed significant differences between the two samples: fibrocytes and precursor T cells in the treatment-sensitive sample exhibited more pronounced changes compared to the treatment-resistant sample, indicating a potential link between precursor T cells, fibrocytes, and therapeutic sensitivity.

Cell-surface ligand-receptor interactions are crucial for intercellular communication. To further investigate these interactions among different cell types, we performed cell-cell interaction (CCI) analysis using stLearn, identifying 10 significant ligand-receptor pairs (Figure 6E). Potential interactions were detected between malignant epithelial cells and precursor T cells, including adhesion junctions (ICAM3-ITGB2, ICAM2-ITGB2, PTPRM-PTPRM) and immune regulation (SPON2-ITGB2). Spatial mapping of significant ligand-receptor pair scores indicated substantial interactions between malignant epithelial cells and precursor T cells through adhesion and immune regulation in the treatment-sensitive sample (Figure 6F). In contrast, CCI analysis of the treatment-resistant sample (No.36) revealed immune regulation (SLAMF7-SLAMF7) and chemokine interactions (CCL11-CCR2) (Figure 6G-H).

### Key Metabolites in Distinct Microregions

**Figure 7:**
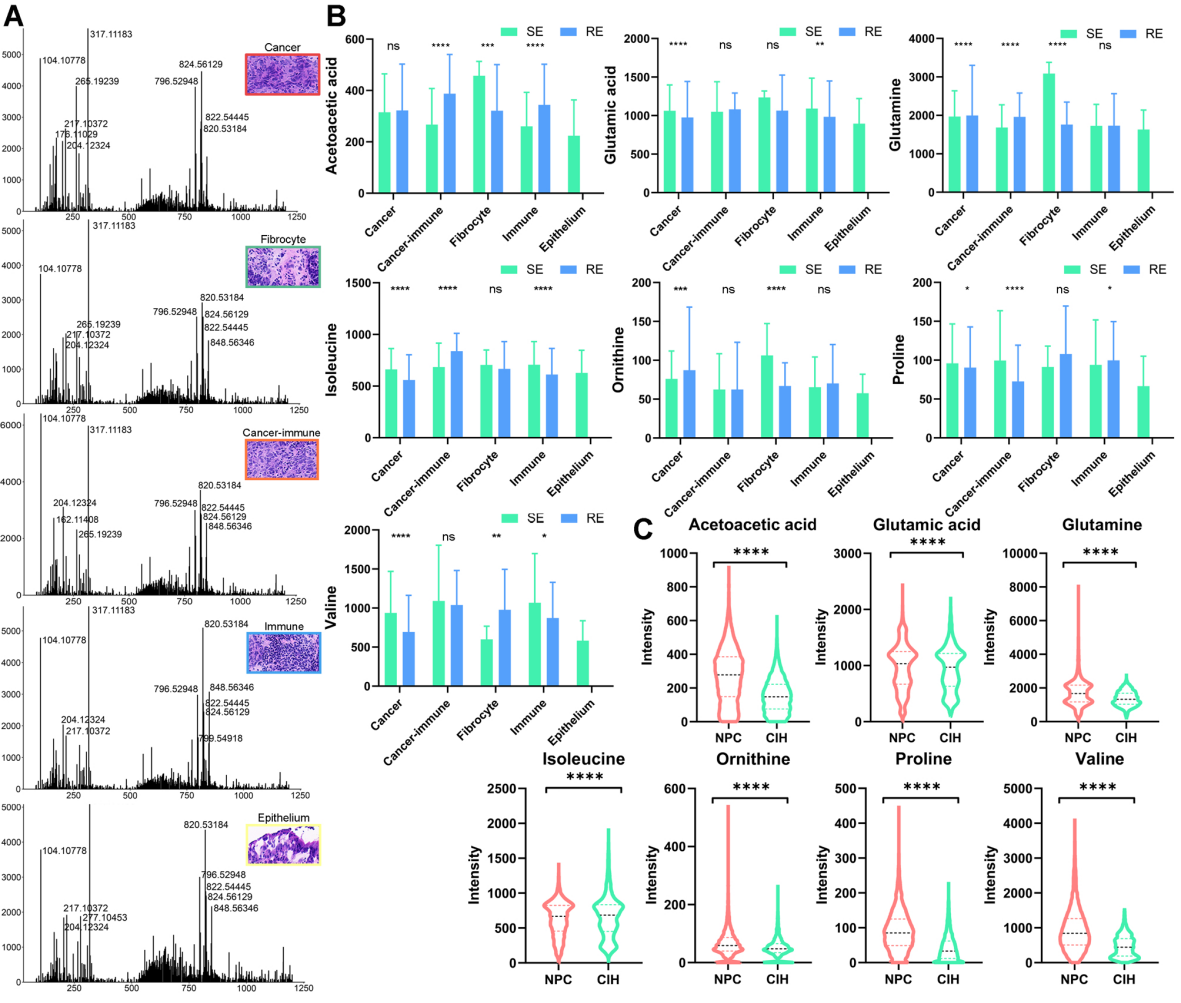
** Microregional Metabolite Expression Analysis.** A: Mass spectra analysis of cancer, fibrocyte, cancer-immune, immune, and epithelium regions. B: Boxplots comparing metabolite expression levels across different regions and groups (treatment-sensitive vs. resistant) using unpaired two-tailed t-tests. C: Boxplots comparing metabolite expression levels in NPC and CIH samples using unpaired two-tailed t-tests. RE: treatment-resistant. SE: treatment-sensitive.

We further examined the expression of key metabolites in various pathological regions of the tumor. Using MsiReader software, we matched selected regions from HE-stained images with spatial metabolomics (SM) ion imaging maps and extracted spectra from specific regions using Waters SYNAPT XS (Figure 7A). We analyzed the expression of key metabolites involved in pathways highlighted in Figure 4, across different regions and groups (Figure 7B). In tumor regions of the treatment-sensitive group, glutamic acid, isoleucine, proline, and valine were expressed at higher levels compared to the treatment-resistant group (p < 0.05). Conversely, glutamine and ornithine were more abundant in the treatment-resistant group. Additionally, metabolites such as acetoacetic acid, glutamic acid, isoleucine, proline, and valine exhibited differences between immune regions across different groups, suggesting that these metabolites may influence the tumor microenvironment and affect therapeutic sensitivity.

We also compared the expression of key metabolites between NPC and CIH samples using SM data (Figure 7C). Apart from isoleucine, which was lower in tumor samples, acetoacetic acid, glutamic acid, glutamine, ornithine, proline, and valine were significantly elevated in NPC. These expression changes highlight significant metabolic reprogramming within the BCAA and glutamate metabolic pathways in NPC.

### Key Genes in Microregions and Clinical Significance Analysis

**Figure 8:**
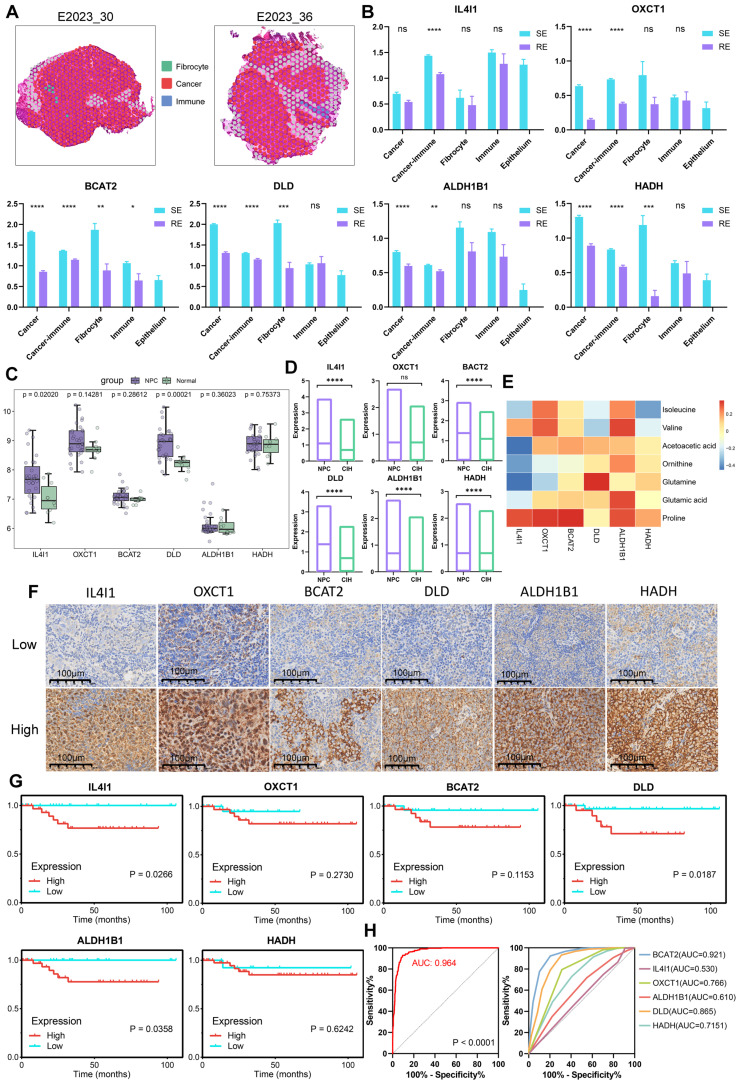
** Spatial Gene Expression and Clinical Significance Analysis.** A: Spatially defined regions on tissue sections from two patients, with different colors representing different regions. B: Boxplots comparing gene expression levels across different regions and groups (treatment-sensitive vs. resistant) using unpaired two-tailed t-tests. C: Comparison of gene expression levels in NPC and normal samples using GEO database and Wilcoxon test. D: Comparison of gene expression levels in NPC and CIH samples using ST data and unpaired two-tailed t-tests. E: Heatmap of metabolite and gene correlations (Pearson coefficient). F: Immunohistochemistry (IHC) technique was employed to demonstrate the expression profiles of six markers in NPC tissues. G: Gene survival curves based on Immunohistochemical data, plotted using GraphPad. H: ROC curves for predicting treatment response (treatment-sensitive vs. resistant), including combined analysis of six indicators (left) and direct calculation of single indicators (right). RE: treatment-resistant. SE: treatment-sensitive.

We mapped pathological regions to ST spots according to their location in HE images, defining the ST spot regions as shown in Figure 8A and Figure S4A. Six key genes involved in BCAA metabolism (*IL4I1, OXCT1, BCAT2, DLD, ALDH1B1, HADH*) were highly expressed in the treatment-sensitive group, with OXCT1, BCAT2, DLD, ALDH1B1 and HADH showing elevated expression in tumor regions (Figure 8B). Group comparisons revealed that these six genes were highly expressed in the cancer-immune regions of the treatment-sensitive group, indicating that BCAA metabolic reprogramming is associated with changes in therapeutic response and the immune microenvironment.

To further assess the diagnostic and therapeutic significance of these key molecules in NPC, we compared the expression levels of the six genes between tumor and normal samples using data from GEO (Figure 8C). All six genes were highly expressed in tumors, with IL4I1 and DLD showing p-values < 0.05. Validation using ST datasets yielded similar results, confirming elevated expression of all six genes in tumors (Figure 8D). We further analyzed the expression profiles of six genes across various cell types and observed differential expression levels among these cells (Figure S4B). Correlating gene and metabolite expression data in corresponding pathological regions revealed strong correlations, underscoring the tight transcriptional and molecular linkage in metabolic reprogramming (Figure 8E).

Finally, we assessed the prognostic impact of these key genes in NPC by conducting survival analysis using Immunohistochemical data. High expression of IL4I1, OXCT1, BCAT2, DLD, ALDH1B1, and HADH was significantly associated with poor prognosis in NPC patients (Figure 8F-G). Furthermore, analysis of head and neck cancer data from the TCGA database showed that although the findings did not reach statistical significance, high expression of these genes similarly correlated with poor patient outcomes (Figure S4C). These results provide strong evidence supporting the potential of these genes as prognostic biomarkers. We also evaluated the ability of these six genes to distinguish between treatment-sensitive and treatment-resistant samples in ST cancer regions. Using logistic regression to combine indicators, we plotted ROC curves to assess the performance of the classification model, which demonstrated a robust AUC of 0.964 (Figure 8H). Additionally, individual gene analysis revealed AUC values of 0.921 for BCAT2 and 0.865 for DLD, suggesting that these six key genes may serve as potential prognostic markers for NPC.

### Functional Implications of Key Genes in NPC

**Figure 9:**
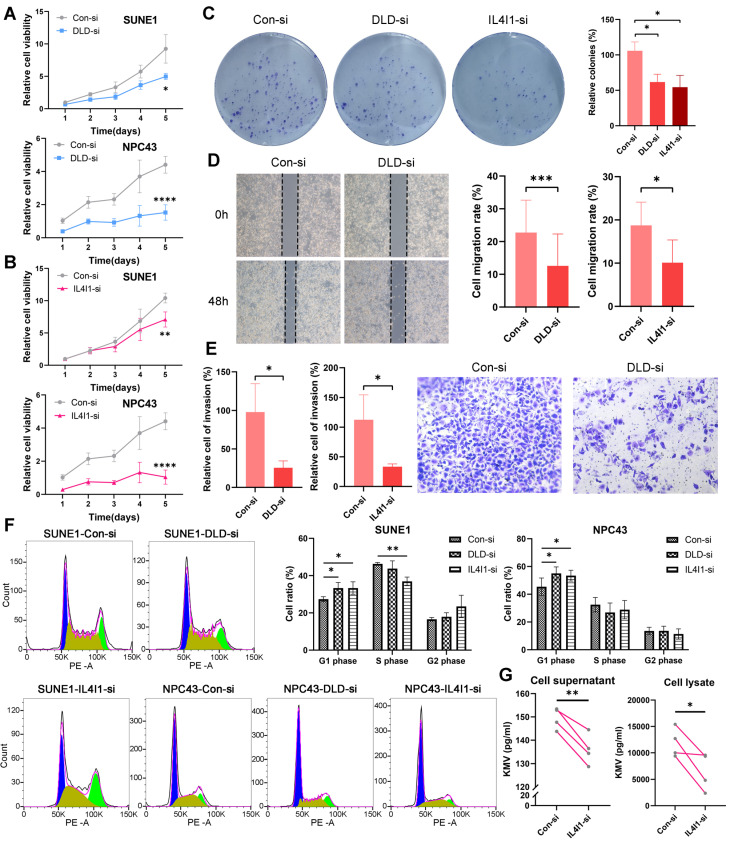
** Knockdown of DLD and IL4I1 promotes proliferation and migration in NPC.** Expression of DLD (A) and IL4I1 (B) was inhibited in SUNE1 and NPC43 cell lines, and cell proliferation was assessed using the CCK-8 assay. C: Colony formation assays were conducted to evaluate the proliferation capacity following DLD and IL4I1 knockdown. D: Wound-healing assays were performed to measure cell migration rates. E: Transwell assays were utilized to evaluate cell invasion ability. F: Flow cytometry was conducted to analyze cell cycle distribution (left), and the percentage of cells in G1, S, and G2 phases was quantified (right). G: ELISA was performed to measure the concentration of the metabolite KMV regulated by IL4I1 in both cell supernatants and lysates after IL4I1 knockdown. *p < 0.05, **p < 0.01, ***p < 0.001, ****p < 0.0001.

In this study, we conducted *in vitro* functional validations for the key genes DLD and IL4I1. The efficiency of knocking down DLD and IL4I1 genes in two cell lines was verified through RT-qPCR technology (Figure S4D). Silencing of the DLD gene using siRNA significantly inhibited the growth of SUNE1 cells and NPC43 cells (Figure 9A), with a similar reduction observed following the silencing of IL4I1 (Figure 9B). Further experiments revealed that knockdown of DLD or IL4I1 markedly impaired the colony-forming ability of NPC cells (Figure 9C), reduced wound healing capacity (Figure 9D), and suppressed cell invasion (Figure 9E).

Cell cycle analysis demonstrated effects on cell cycle arrest: silencing DLD and IL4I1 primarily caused G1 phase arrest (Figure 9F). Moreover, metabolic profiling indicated reduced KMV levels upon IL4I1 knockdown, underscoring the critical roles of these genes in cellular metabolic regulation (Figure 9G).

## Discussion

In this study, we utilized spatial metabolomics (SM) and spatial transcriptomics (ST) to investigate the metabolic factors associated with treatment response and PD-1 expression in NPC. Our objective was to generate new insights that could inform therapeutic response. Previous studies have highlighted metabolic molecules linked to therapeutic resistance in NPC 16, 17, yet the specific metabolic pathways and alterations in relevant metabolites and enzymes involved in therapeutic resistance remain poorly understood.

SM enables the spatial mapping of metabolic ion distribution and expression, and has been successfully applied to various cancer types. For instance, SM has revealed four distinct metabolic patterns in different lung cancer subtypes, thereby improving the classification of lung cancer 18. Similarly, SM has been used to characterize metabolic profiles in renal and gastric cancers 19, 20. Spatial lipidomics has also proven valuable in identifying safe surgical margins for oral squamous cell carcinoma 21. However, the spatial metabolic features of NPC remain largely unexplored.

Metabolites such as glucose, linoleic acid, stearic acid, arachidonic acid, proline, β-hydroxybutyric acid, and 1-hexadecanoylglycerol have been shown to effectively differentiate NPC patients from normal controls 22. Additionally, superoxide dismutase 1 (SOD1), which is upregulated in NPC, enhances fatty acid oxidation via carnitine palmitoyltransferase 1A (CPT1A), presenting a potential prognostic marker for the disease 23. While much of the metabolomic research on NPC has focused on individual metabolites or enzymes, our study integrates SM and ST to examine treatment response-related metabolic molecules at the spatial level, linking regulatory genes with metabolites, thus providing insights into changes in broader metabolic pathways.

Our results demonstrate that NPC patients with therapeutic sensitivity or high PD-1 expression exhibit more active branched-chain amino acid (BCAA) metabolism, glutamine metabolism, and lipid metabolism. Alterations in BCAA metabolism have been observed in several cancers 24. Wen *et al.* (2018) showed that BCAA transaminase 1 (BCAT1), a key enzyme in BCAA catabolism, is overexpressed in NPC and promotes cell proliferation, making it a promising molecular target for NPC treatment 25. In breast cancer, BCAT1 has been linked to mTOR signaling, enhancing mitochondrial function and cancer cell growth 26. Our study further corroborates the relationship between BCAA metabolism and therapeutic efficacy in NPC.

Glutamine metabolism is crucial for tumorigenesis and chemoresistance in cancers 27. The expression of glutamine synthase (GS) influences hepatocellular carcinoma's sensitivity to chemoradiotherapy 28. Moreover, targeting glutamine metabolism has been shown to enhance the effectiveness of anti-PD-1/PD-L1 therapies 29. Additionally, lipid metabolic reprogramming has been implicated in resistance to chemotherapy and radiotherapy in tumors 30, while boosting lipid metabolism has been found to enhance immunogenicity and improve melanoma immunotherapy outcomes 31. In our study, we observed strong associations between glutamine metabolism, lipid metabolism, and therapeutic efficacy in NPC.

ST, which retains the spatial context of cells within tissue samples, provides valuable insights into the tumor microenvironment. In this study, we utilized ST not only to visualize the spatial expression of genes regulating metabolism but also to investigate molecular events, cell types, and interactions related to treatment response and PD-1 expression. Our analysis of cell proportions revealed that precursor T cells and macrophages were associated with treatment response and PD-1 expression. Macrophages play dual roles in chemoradiotherapy, both enhancing anti-tumor effects and limiting efficacy through tissue repair coordination 32. Larroquette *et al.* found that macrophage enrichment in non-small cell lung cancer correlates with resistance to anti-PD-1/PD-L1 immunotherapy 33. Moreover, our neighbor analysis suggests that fibroblasts may contribute to therapeutic efficacy and PD-1 expression. The relationship between the tumor microenvironment and therapy response has been well-documented 34, 35, with Kieffer *et al.* identifying a fibroblast subpopulation linked to immunotherapy resistance 36. These findings suggest that specific macrophage and fibroblast subpopulations may contribute to both chemo-radiotherapy and immunotherapy resistance in NPC, presenting potential therapeutic targets.

Furthermore, we identified six key genes—*IL4I1, OXCT1*, *BCAT2*, *DLD*, *ALDH1B1*, and *HADH*—and developed a classification model combining these markers to predict therapeutic response. This model achieved an AUC of 0.964, demonstrating a strong association between these genes and therapeutic sensitivity. IL4I1 is a tryptophan-metabolizing enzyme that depletes tryptophan and generates immunomodulatory metabolites in tumor microenvironment 37. In our study, IL4I1 was highly expressed in tumors and was notably more abundant in the cancer-immune zone of treatment-sensitive samples, consistent with its role in promoting tumor progression and suppressing anti-tumor immunity 38. OXCT1, a key enzyme in ketone metabolism 39, was also highly expressed in tumor cells, suggesting its involvement in tumor immunity and treatment response. BCAT2, which regulates BCAA catabolism, was upregulated in treatment-sensitive samples, and its role in modulating the tumor microenvironment suggests it could impact therapeutic sensitivity 40. DLD is a component of the branched-chain amino acid-dehydrogenase complex and is involved in the catabolism of branched-chain amino acids 41. ALDH1B1, a mitochondrial NAD+-dependent enzyme, is involved in detoxifying aldehydes and maintaining cellular redox balance, thereby supporting cell survival and potentially contributing to chemoresistance 42. HADH, a member of the 3-hydroxyacyl-CoA dehydrogenase gene family, is a potential prognostic predictor in malignant lymphoma 43. The involvement of DLD, ALDH1B1, and HADH in these essential metabolic pathways, along with their associations with treatment response 42-44, highlights their potential as prognostic biomarkers in NPC, offering valuable insights for clinical management and personalized therapy.

This study has several limitations. First, the sample size, while reasonable, remains limited, and larger, more diverse cohorts are needed to enhance statistical power and generalizability. Another limitation is the potential impact of metabolite stability during sample collection, processing, and storage. Variations in metabolite levels due to improper storage or handling could affect the accuracy and reproducibility of the results. Similarly, the sensitivity of the analytical methods employed, particularly in detecting low-abundance metabolites or genes, could introduce variability in the data. Finally, while we identified key genes linked to treatment response, further functional validation of these genes *in vivo* is necessary to establish their roles in therapeutic resistance and confirm causality.

In conclusion, this study integrates spatial metabolomics and spatial transcriptomics to shed light on the metabolic landscape of NPC. Our findings emphasize the crucial role of BCAA metabolism, glutamine metabolism, and lipid metabolism in therapeutic response and PD-1 expression. By identifying key metabolic pathways and genes, we developed a robust classification model with high predictive power for distinguishing between treatment-sensitive and treatment-resistant cases. These results not only contribute to the academic understanding of NPC but also hold significant potential for clinical application in personalized treatment strategies, ultimately improving patient outcomes.

## Supplementary Material

Supplementary figures and table.
