# Supplementary material for: Spatial Metabolomics and Transcriptomics Reveal Metabolic Reprogramming and Cellular Interactions in Nasopharyngeal Carcinoma with High PD-1 Expression and Therapeutic Response
Source: Theranostics. 2025 Feb 10;15(7):3035–54. doi: 10.7150/thno.102822 (PMC11898293; doi:10.7150/thno.102822)
Supplement: Supplementary file 1 — Supplementary figures and table. [file thnov15p3035s1.pdf]

Figure S1. Spatial Analysis of Gene and UMI Expression. A: HE stained images of tissue sections from an NPC patient, including cancer-immune and epithelium regions. B: Top: Violin plot showing the number of genes expressed per spot in the section. Bottom: Expression levels of gene numbers across the spatial section, with colors from green to yellow to red indicating increasing numbers of expressed genes per spot. C: Top: Expression levels of UMI numbers across the spatial section, with colors from green to yellow to red indicating increasing UMI numbers per spot. Bottom: Violin plot showing the number of UMIs per spot. D: UMAP clustering of spots by sample, with different colors representing different samples.

Figure S2. Metabolite Analysis and Model Evaluation. A: Mass spectra analysis of samples. B: Evaluation of OPLS-DA model overfitting. Criteria: 1. All left-side green Q2 values are lower than the original right-side points; 2. The green regression line of the Q2 points intersects the y-axis (left side) at or below zero. C: Annotation of cell composition in spatial spots using the RCTD algorithm, showing the top two cell types for each spot in two samples.

Figure S3. GSVA Enrichment Analysis and Cell Type Correlation. A: GSVA enrichment analysis of all spots in spatial tissue sections from six NPC samples, presented as a heatmap of signaling pathways. The background gene set is the KEGG database. The x-axis represents sample names and groups, and the y-axis represents metabolic entries. B: Heatmap of cell type correlations (Pearson coefficient) by group, with both axes representing cell types. C: Differential expression of transition marker genes during the spatial trajectory differentiation of subcluster 1. Genes increasing along this trajectory are shown in red (upregulated), and those decreasing are shown in blue (downregulated). D: Visualization of metascape enrichment analysis results for the transition gene set. E: Spatial presentation of specific cell types (malignant epithelial cells, precursor T cells) in different samples across four hierarchical regions. The distance from the reference cell type is divided into four levels: reference (0), start (1/3 quantile distance), middle (2/3 quantile distance), and end (farthest).

Figure S4. Analysis of pathological regions, gene expression, survival curves, and gene knockdown effects. A: Pathological regions identified in spatial slices from patient No. 43 are delineated. B: Expression patterns of genes across nine major cell types are shown based on ST data. C: Survival curves were generated using data from the TCGA database. D: The expression levels of DLD and IL4I1 were assessed via Q-RT-PCR following their knockdown in SUNE1 (left) and NPC43 (right) cell lines.

Figure S1

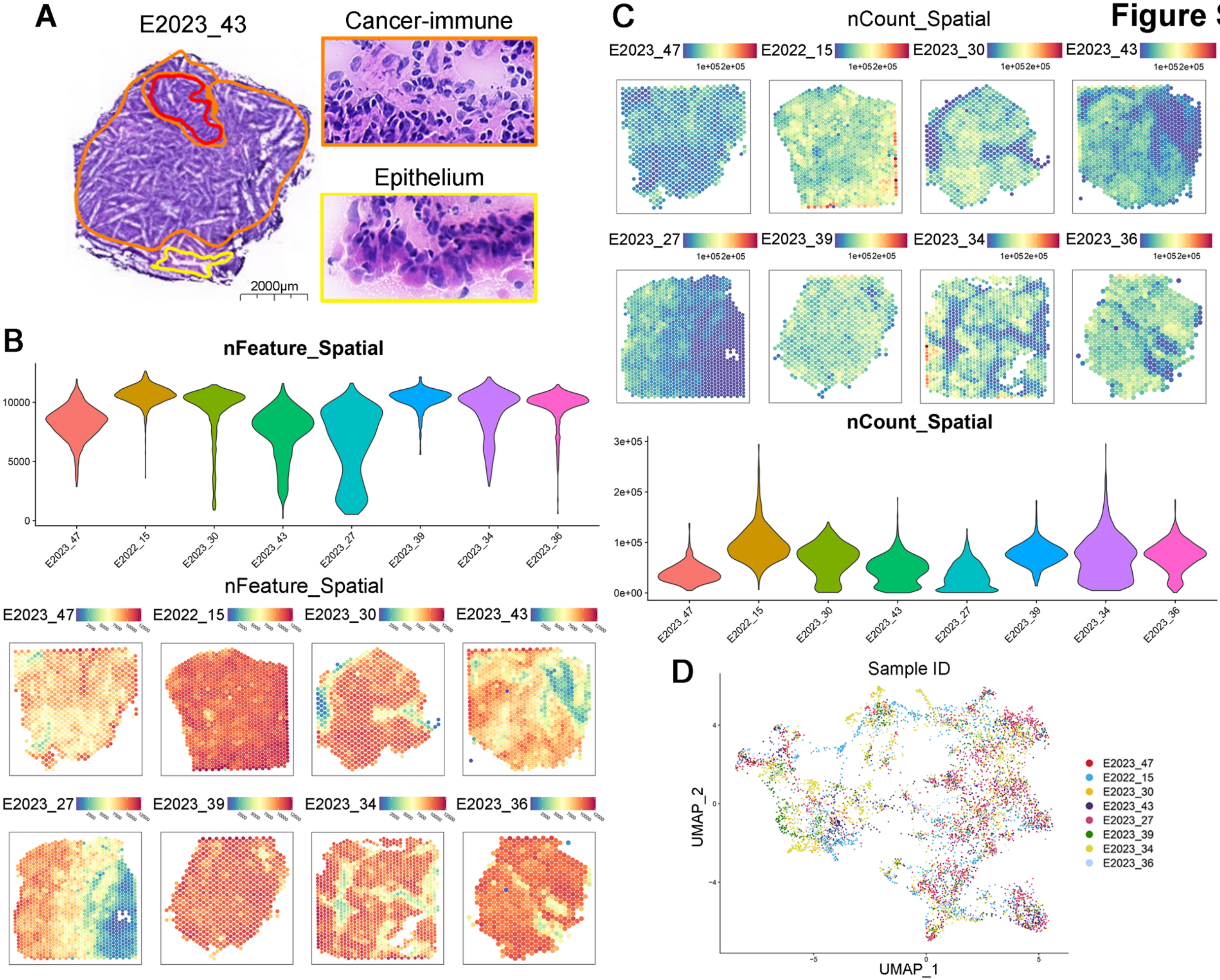
A
E2023_43
Cancer-immune
Epithelium
2000μm
B
nFeature_Spatial
10000
5000
0
E2023_47
E2022_15
E2023_30
E2023_43
E2023_27
E2023_39
E2023_34
E2023_36
nFeature_Spatial
C
nCount_Spatial
1e+052e+05
nCount_Spatial
3e+05
2e+05
1e+05
0e+00
D
Sample ID
UMAP_1
UMAP_2

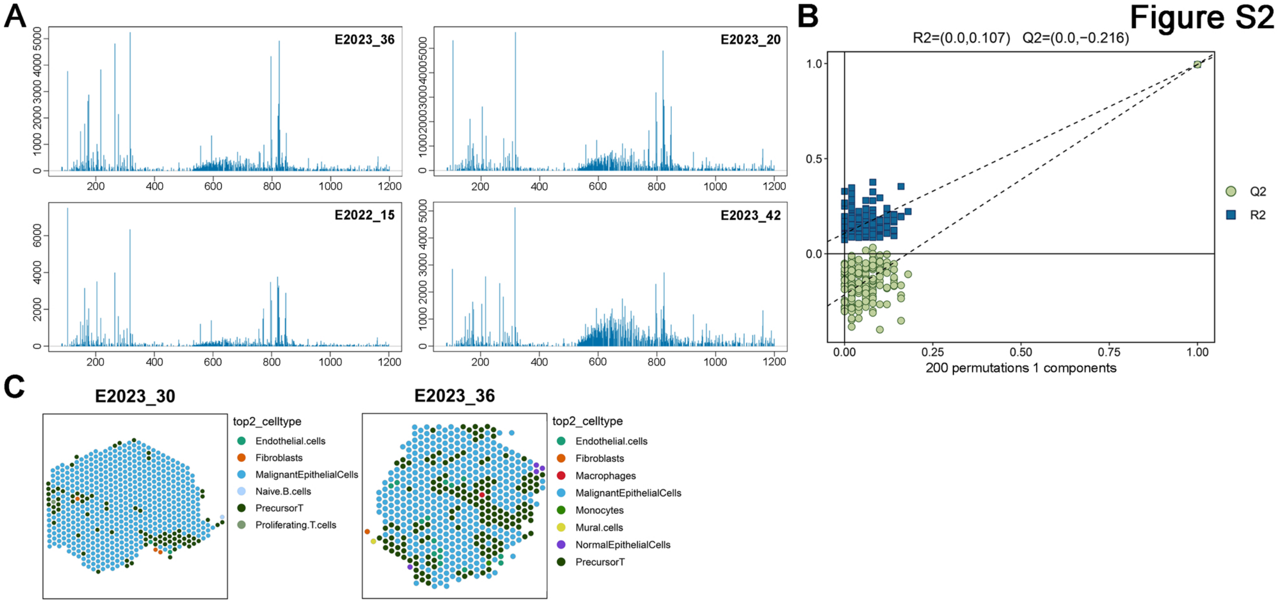
Figure S2
A
E2023_36
E2023_20
E2022_15
E2023_42
B
R2=(0.0,0.107) Q2=(0.0,−0.216)
Q2
R2
200 permutations 1 components
C
E2023_30
E2023_36
top2_celltype
Endothelial.cells
Fibroblasts
MalignantEpithelialCells
Naive.B.cells
PrecursorT
Proliferating.T.cells
top2_celltype
Endothelial.cells
Fibroblasts
Macrophages
MalignantEpithelialCells
Monocytes
Mural.cells
NormalEpithelialCells
PrecursorT

Figure S3

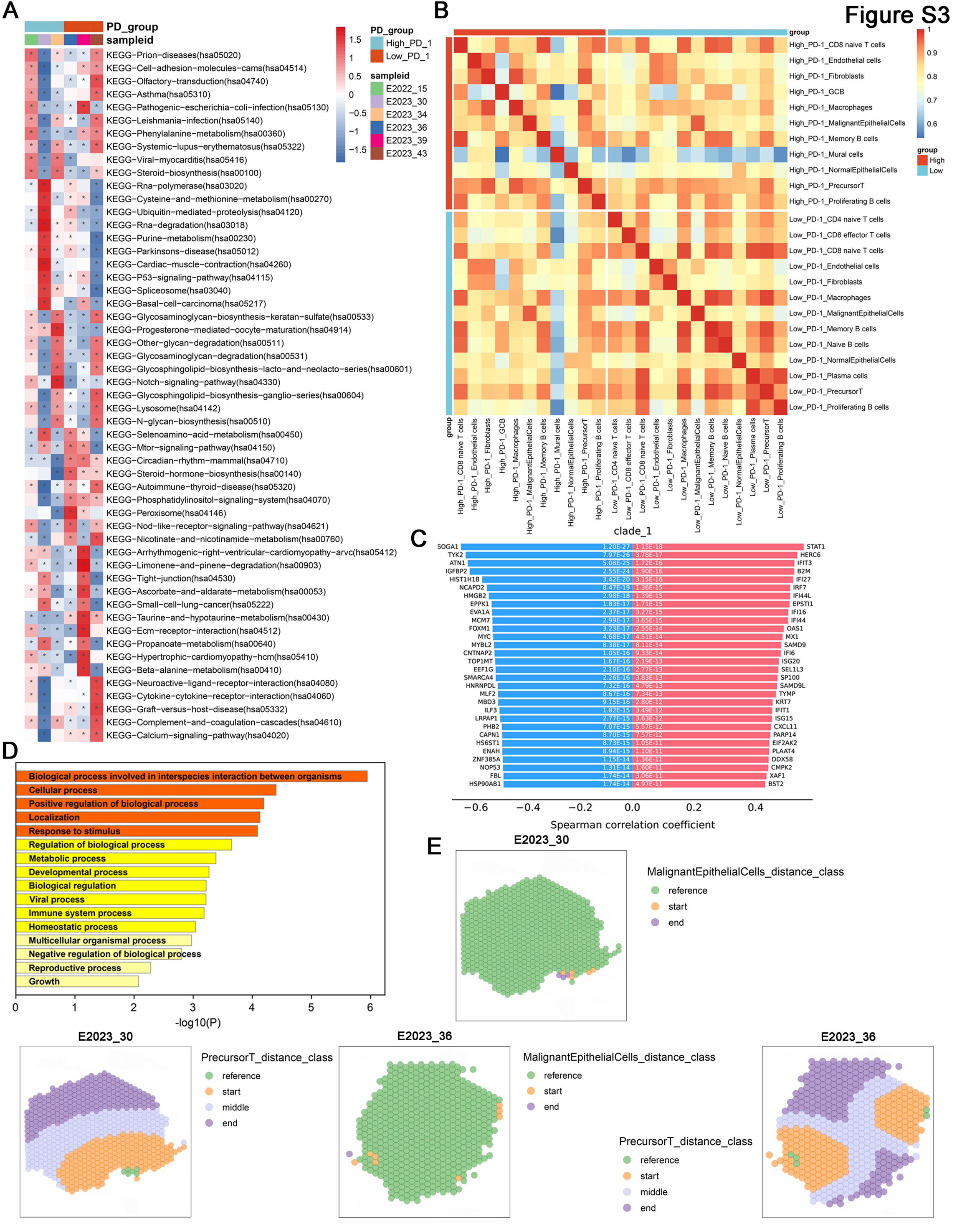
A
PD_group
sampleid
High_PD_1
Low_PD_1
E2022_15
E2023_30
E2023_34
E2023_36
E2023_39
E2023_43
1.5
1
0.5
0
−0.5
−1
−1.5
KEGG−Prion−diseases(hsa05020)
KEGG−Cell−adhesion−molecules−cams(hsa04514)
KEGG−Olfactory−transduction(hsa04740)
KEGG−Asthma(hsa05310)
KEGG−Pathogenic−escherichia−coli−infection(hsa05130)
KEGG−Leishmania−infection(hsa05140)
KEGG−Phenylalanine−metabolism(hsa00360)
KEGG−Systemic−lupus−erythematosus(hsa05322)
KEGG−Viral−myocarditis(hsa05416)
KEGG−Steroid−biosynthesis(hsa00100)
KEGG−Rna−polymerase(hsa03020)
KEGG−Cysteine−and−methionine−metabolism(hsa00270)
KEGG−Ubiquitin−mediated−proteolysis(hsa04120)
KEGG−Rna−degradation(hsa03018)
KEGG−Purine−metabolism(hsa00230)
KEGG−Parkinsons−disease(hsa05012)
KEGG−Cardiac−muscle−contraction(hsa04260)
KEGG−P53−signaling−pathway(hsa04115)
KEGG−Spliceosome(hsa03040)
KEGG−Basal−cell−carcinoma(hsa05217)
KEGG−Glycosaminoglycan−biosynthesis−keratan−sulfate(hsa00533)
KEGG−Progesterone−mediated−oocyte−maturation(hsa04914)
KEGG−Other−glycan−degradation(hsa00511)
KEGG−Glycosaminoglycan−degradation(hsa00531)
KEGG−Glycosphingolipid−biosynthesis−lacto−and−neolacto−series(hsa00601)
KEGG−Notch−signaling−pathway(hsa04330)
KEGG−Glycosphingolipid−biosynthesis−ganglio−series(hsa00604)
KEGG−Lysosome(hsa04142)
KEGG−N−glycan−biosynthesis(hsa00510)
KEGG−Selenoamino−acid−metabolism(hsa00450)
KEGG−Mtor−signaling−pathway(hsa04150)
KEGG−Circadian−rhythm−mammal(hsa04710)
KEGG−Steroid−hormone−biosynthesis(hsa00140)
KEGG−Autoimmune−thyroid−disease(hsa05320)
KEGG−Phosphatidylinositol−signaling−system(hsa04070)
KEGG−Peroxisome(hsa04146)
KEGG−Nod−like−receptor−signaling−pathway(hsa04621)
KEGG−Nicotinate−and−nicotinamide−metabolism(hsa00760)
KEGG−Arrhythmogenic−right−ventricular−cardiomyopathy−arvc(hsa05412)
KEGG−Limonene−and−pinene−degradation(hsa00903)
KEGG−Tight−junction(hsa04530)
KEGG−Ascorbate−and−aldarate−metabolism(hsa00053)
KEGG−Small−cell−lung−cancer(hsa05222)
KEGG−Taurine−and−hypotaurine−metabolism(hsa00430)
KEGG−Ecm−receptor−interaction(hsa04512)
KEGG−Propanoate−metabolism(hsa00640)
KEGG−Hypertrophic−cardiomyopathy−hcm(hsa05410)
KEGG−Beta−alanine−metabolism(hsa00410)
KEGG−Neuroactive−ligand−receptor−interaction(hsa04080)
KEGG−Cytokine−cytokine−receptor−interaction(hsa04060)
KEGG−Graft−versus−host−disease(hsa05332)
KEGG−Complement−and−coagulation−cascades(hsa04610)
KEGG−Calcium−signaling−pathway(hsa04020)
B
group
High_PD-1_CD8 naive T cells
High_PD-1_Endothelial cells
High_PD-1_Fibroblasts
High_PD-1_GCB
High_PD-1_Macrophages
High_PD-1_MalignantEpithelialCells
High_PD-1_Memory B cells
High_PD-1_Mural cells
High_PD-1_NormalEpithelialCells
High_PD-1_PrecursorT
High_PD-1_Proliferating B cells
Low_PD-1_CD4 naive T cells
Low_PD-1_CD8 effector T cells
Low_PD-1_CD8 naive T cells
Low_PD-1_Endothelial cells
Low_PD-1_Fibroblasts
Low_PD-1_Macrophages
Low_PD-1_MalignantEpithelialCells
Low_PD-1_Memory B cells
Low_PD-1_Naive B cells
Low_PD-1_NormalEpithelialCells
Low_PD-1_Plasma cells
Low_PD-1_PrecursorT
Low_PD-1_Proliferating B cells
1
0.9
0.8
0.7
0.6
group
High
Low
clade_1
C
SOGA1 1.20E-27 1.15E-18 STAT1
TYK2 7.97E-26 3.78E-17 HERC6
ATN1 5.08E-25 1.72E-16 IFIT3
IGFBP2 2.55E-24 1.90E-16 B2M
HIST1H1B 3.42E-20 3.15E-16 IFI27
NCAPD2 8.47E-19 1.36E-15 IRF7
HMGB2 2.98E-18 1.39E-15 IFI44L
EPPK1 1.83E-17 1.71E-15 EPSTI1
EVA1A 2.37E-17 3.27E-15 IFI16
MCM7 2.99E-17 3.65E-15 IFI44
FOXM1 3.23E-17 2.55E-14 OAS1
MYC 4.68E-17 4.51E-14 MX1
MYBL2 8.38E-17 8.11E-14 SAMD9
CNTNAP2 1.05E-16 9.33E-14 IFI6
TOP1MT 1.67E-16 2.19E-13 ISG20
EEF1G 2.10E-16 2.77E-13 SEL1L3
SMARCA4 2.26E-16 3.83E-13 SP100
HNRNPDL 7.32E-16 4.79E-13 SAMD9L
MLF2 8.67E-16 7.34E-13 TYMP
MBD3 9.15E-16 2.80E-12 KRT7
ILF3 1.82E-15 3.49E-12 IFIT1
LRPAP1 2.77E-15 3.63E-12 ISG15
PHB2 7.07E-15 5.57E-12 CXCL11
CAPN1 8.70E-15 7.57E-12 PARP14
HS6ST1 8.73E-15 1.05E-11 EIF2AK2
ENAH 8.94E-15 1.10E-11 PLAAT4
ZNF385A 1.15E-14 1.36E-11 DDX58
NOP53 1.31E-14 1.60E-11 CMPK2
FBL 1.74E-14 3.06E-11 XAF1
HSP90AB1 1.74E-14 4.97E-11 BST2
−0.6 −0.4 −0.2 0.0 0.2 0.4
Spearman correlation coefficient
D
Biological process involved in interspecies interaction between organisms
Cellular process
Positive regulation of biological process
Localization
Response to stimulus
Regulation of biological process
Metabolic process
Developmental process
Biological regulation
Viral process
Immune system process
Homeostatic process
Multicellular organismal process
Negative regulation of biological process
Reproductive process
Growth
0 1 2 3 4 5 6
-log10(P)
E
E2023_30
MalignantEpithelialCells_distance_class
reference
start
end
E2023_30
PrecursorT_distance_class
reference
start
middle
end
E2023_36
MalignantEpithelialCells_distance_class
reference
start
end
PrecursorT_distance_class
reference
start
middle
end
E2023_36

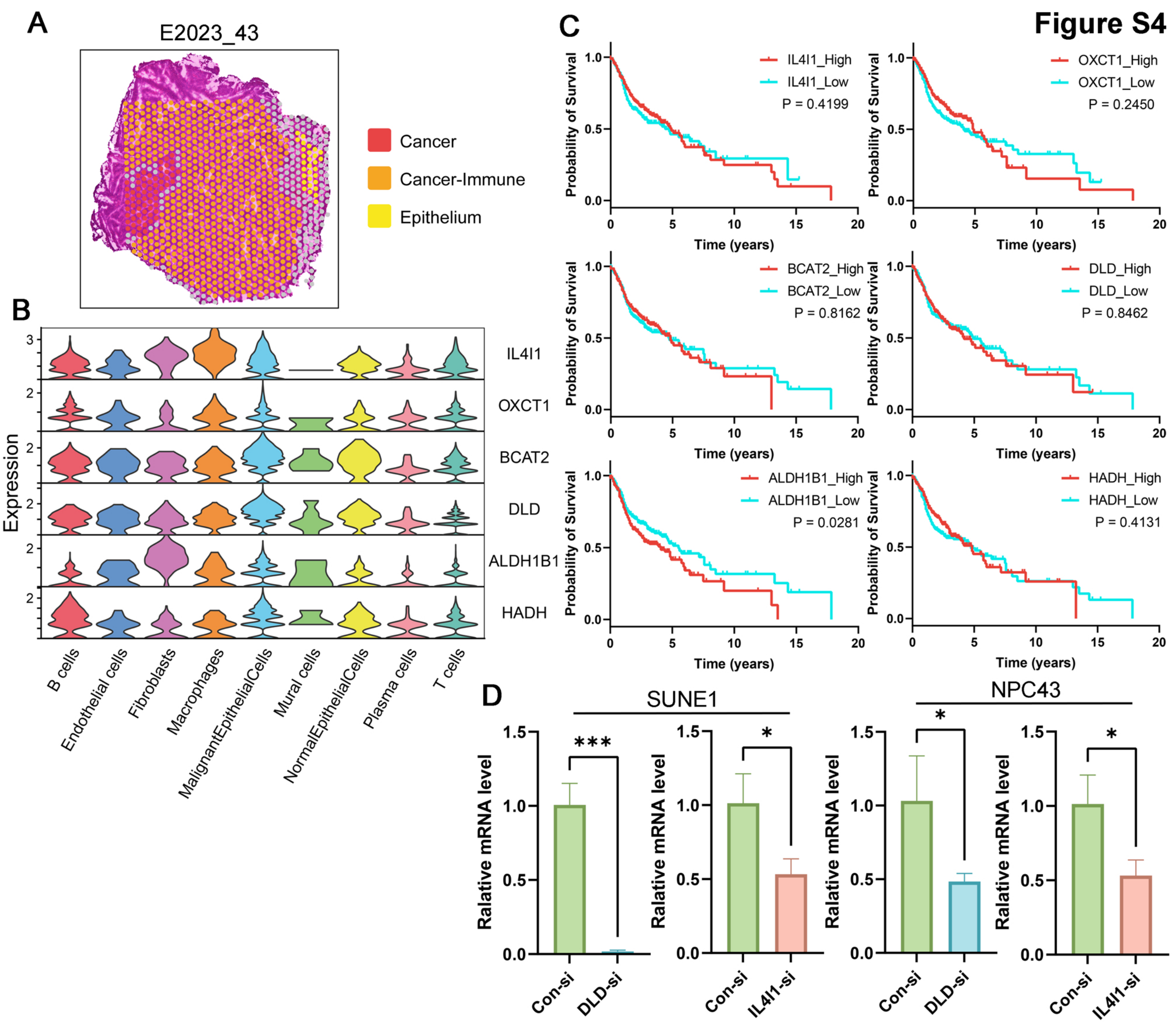
Figure S4
A
E2023_43
Cancer
Cancer-Immune
Epithelium
B
Expression
IL4I1
OXCT1
BCAT2
DLD
ALDH1B1
HADH
B cells
Endothelial cells
Fibroblasts
Macrophages
MalignantEpithelialCells
Mural cells
NormalEpithelialCells
Plasma cells
T cells
C
Probability of Survival
Time (years)
IL4I1_High
IL4I1_Low
P = 0.4199
OXCT1_High
OXCT1_Low
P = 0.2450
BCAT2_High
BCAT2_Low
P = 0.8162
DLD_High
DLD_Low
P = 0.8462
ALDH1B1_High
ALDH1B1_Low
P = 0.0281
HADH_High
HADH_Low
P = 0.4131
D
SUNE1
NPC43
Ralative mRNA level
***
*
*
*
Con-si
DLD-si
Con-si
IL4I1-si
Con-si
DLD-si
Con-si
IL4I1-si

Table S1. Characteristics of patients

Patient	ST*	Gender	Age	Dignosis	PD-1 group	TNM
No.27	1	M	26	CIH	NA	NA
No.31	0	M	37	CIH	NA	NA
No.35	0	F	36	CIH	NA	NA
No.37	0	M	36	CIH	NA	NA
No.47	1	M	23	CIH	NA	NA
No.15	1	M	38	NPC	High	T4N2M0
No.18	0	F	52	NPC	Low	T3N2M0
No.19	0	M	14	NPC	Low	T3N3M0
No.20	0	F	77	NPC	High	T2N2M0
No.30	1	M	69	NPC	High	T4N1M0
No.34	1	M	60	NPC	High	T4N2M0
No.36	1	M	53	NPC	Low	T3N3M0
No.39	1	M	65	NPC	Low	T4N3M0
No.42	0	F	45	NPC	Low	T3N2M0
No.43	1	M	65	NPC	Low	T2N1M0
No.46	0	M	37	NPC	High	T3N2M0

*1: the sample underwent spatial transcriptomics (ST) analysis. 0: the sample did not undergo ST analysis.
